# An illustrated guide to the identification of the known species of *Diatraea* Guilding (Lepidoptera, Crambidae, Crambinae) based on genitalia

**DOI:** 10.3897/zookeys.565.6797

**Published:** 2016-02-17

**Authors:** M. Alma Solis, Mark A. Metz

**Affiliations:** 1 Systematic Entomology Laboratory, Beltsville Agriculture Research Center, Agricultural Research Service, U.S. Department of Agriculture, c/o National Museum of Natural History, E-517, MRC 168, Smithsonian Institution, PO Box 37012, Washington, DC 20013-7012, U.S.A.

**Keywords:** *Diatraea*, sugarcane moths, Poaceae, biofuels, genitalia

## Abstract

The genus *Diatraea* Guilding is one of the most economically important groups of moths in the Western Hemisphere. The larvae are stem borers that feed on species of Poaceae, or grasses, such as sugarcane, corn, rice, and sorghum, as well as many other native grasses. Interest in this group has risen considerably since sugarcane and other grasses have been utilized and/or investigated as biofuels. This is the first modern study to treat all 41 valid described species. Most type specimens were examined and we provide a checklist with 19 new synonyms. We provide keys for the identification of most species in this genus based on morphology of the male and female genitalia and modern illustrations of male and female genitalia. We also provide an updated table of species distribution by country.

## Introduction

**Figure 1.**
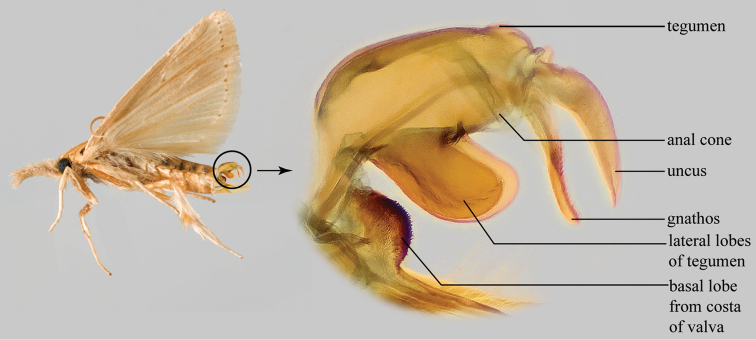
Lateral habitus and male genitalia of *Diatraea saccharalis* (Fabricius, 1794), the type species of *Diatraea* (modified from Solis and Metz 2011).

The genus *Diatraea* Guilding is composed of externally similar species, i.e. species cannot be identified using external characters only, and occur in the Western Hemisphere. The type species is *Diatraea saccharalis* (Fabricius, 1794) (Fig. 1), a major pest of sugarcane. The literature is abundant with studies on the biology of this and other closely related species that are economically important beginning with Guilding (1828). In this paper we consider 41 distinct taxa represented by 41 valid names and 46 synonyms. Some synonyms (e.g., *Diatraea busckella* Dyar & Heinrich, 1927) at one time were considered valid species or subspecies based on insignificant amounts of variation and/or locally disparate distributions. Fortunately, the morphology of the genitalia has provided excellent characters for identification for most species. This study treats the entire genus as it is currently circumscribed throughout the Western Hemisphere. We provide a table of the species distributions as is currently known (Suppl. material 1) compiled from Box (1931) and the USNM collection (National Museum of Natural History, Smithsonian Institution, Washington, DC). Absence of a country from this table may not indicate that it does not occur there; it indicates that we have not seen material of that species from that country. During the course of this study we discovered more new species and the potential for cryptic species (e.g. Joyce et al. 2014, Solis et al. 2015), but we decided to publish keys to the identification of described species due to the number of identification requests and workshop requests that were being submitted to MAS (e.g. Solis 2004, Vargas et al. 2013). We provide a key to adults of the Crambinae as modified from Munroe and Solis (1998). The *Diatraea* diagnosis is from an excellent study of the North American Crambinae by Landry (1995).

Over eighty species names have been associated with *Diatraea* or related genera since Fabricius described the type species as *Phalaena saccharalis* in 1794. Early studies by Dyar and Heinrich (1927) and Box (1931) listed 39 and 48 species, respectively. More modern checklists list more, various numbers of species. Bleszynski (1967) lists 55 species, Munroe et al. (1995) lists 57 species, and Nuss et al. (2015) list 58 species. The first overview of *Diatraea* and related genera was in the 1927 treatment by Dyar & Heinrich. They treated the 22 known species and described 9 new species; they created entirely new species concepts. Several species names were unrecognized, that is, their only reference was a species description and not specimens. They were the first to comparatively use and illustrate the male and female genitalia using pen and ink. The last major overview of *Diatraea* and related genera was Box (1931). He recognized 48 species, including 10 new species. He illustrated the genitalia with black and white photos when he deemed the pen and ink illustrations deficient. Unfortunately, his photographs were often with insufficient magnification. For the first time, he provided a distribution chart by country for the 48 species. He provided a key to external characters primarily using the frons, forewing color, and venation, although he suggested that genitalia dissections be done whenever possible.

## Methods

Type specimens of *Diatraea* species were studied to confirm identity of species. Approximately 50% of the types are located at the Natural History Museum (BMNH) in London, United Kingdom, and most of the others are located at the National Museum of Natural History (USNM) in Washington, DC, USA. Almost all of the type specimens at the USNM had been previously dissected for the study by Dyar and Heinrich (1927). Dissections of material from the Carnegie Museum of Natural History (CMNH), Pittsburgh, PA, USA, were labeled CMNH and given sequential numbers.

**Figure 2.**
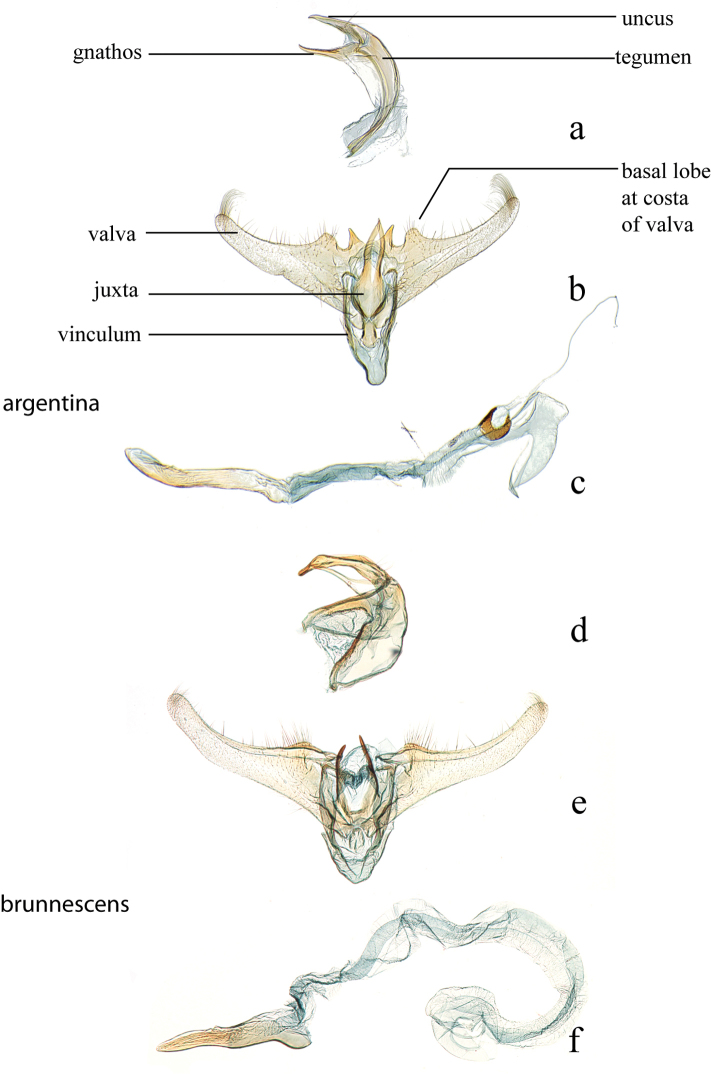
Male genitalia: *Diatraea argentina*, CMNH #002, Santa Cruz, Provincia del Sara, Bolivia **a** lateral view uncus, gnathos, tegumen **b** ventral view vinculum, juxta, valvae **c** phallus; *Diatraea brunnescens*, USNM #114612, New Bremen, Brazil **d** lateral view uncus, gnathos, tegumen **e** ventral view vinculum, juxta, valvae **f** phallus.

Genitalia preparation for identification (Clarke 1941, Robinson 1976): the abdomen is removed by pushing the abdomen up with forceps. If the metathorax is still attached to the abdomen it should be separated from the abdomen. The male or female abdomen is then placed in a vial with 10% + KOH. The vial is then placed in a beaker with boiling water. The abdomen in KOH is boiled until air bubbles can be seen in the abdomen. Alternatively, the abdomen can be left in cold 10% KOH overnight. The abdomen is then removed from the KOH and placed in water. Then a brush is used to clean the scales from the abdomen, particularly the anal area if it is a male. For males the genitalia can be removed by holding the valvae and uncus with forceps, and then pulling posteriorly at the same time that the abdomen is being held anteriorly with either the brush or the forceps. The male genitalia of *Diatraea* can be very sclerotized so often staining is not required to see the structures. At this point, when the genitalia is in water, the male can be identified using the key to males below. A pair of forceps can force the valvae apart or a small piece of glass can be placed on the genitalia to flatten it out to be able to see certain structures. For structure recognition in the male genitalia, two views, lateral (Fig. 1) and flattened (Fig. 2), are given and labeled.

To remove the female genitalia, the abdomen should be cut laterally the entire length and then around the abdomen between segments VI and VII. The female may have more tissues surrounding the genitalia and must be cleaned with a brush carefully. The corpus bursae varies in length and width, so it is better to examine it when it is still turgid. If slide mounted, care should be taken to not fold, tear or collapse the corpus bursae. The female apparatus is usually membranous, but may not need staining. If staining is required to see structures use a saturated solution of chlorazol black for only a few seconds. The female can then be identified using the key to females below. The genitalia can then be stored in genitalia vials with glycerin if available, or in 70% alcohol that may harden, but will also preserve, the genitalia as vouchers. Two videos are also available to view dissection techniques in great detail (Brown et al. 2009, 2011).

Terminology within the keys and the major structures of the male genitalia are as follows (Figs 1, 2): valva (e) (harpe of Dyar and Heinrich 1927), basal lobe from the costa of the valvae, juxta (anellus of Dyar and Heinrich 1927), gnathos, tegumen, lateral lobe of the tegumen (but see Landry 1995, p. 69, “a pair of extensions posterad from base of ventral margin”), vinculum, and the phallus (aedoeagus of Dyar and Heinrich 1927) that includes a vesica with a cornutus (or cornuti).

The female genitalia (Figs 27, 29) consists of the papillae anales or ovipositor, anterior and posterior apophyses, an ostium bursae (genital opening of Dyar and Heinrich 1927), ductus bursae, and corpus bursae; bursae copulatrix is the term used for the ductus bursae + corpus bursae. Associated with the corpus bursae in a few *Diatraea* species is a sclerotized signum or many signa that may take various forms and may be diagnostic of species. Associated with the ostium bursae are: sterigma (= ostiolar sclerites (Gaskin 1971)), sclerotized structures, sometimes very complex, surrounding the ostium bursae; lamella antevaginalis, the anterior, often the ventral, side, of the sterigma; lamella postvaginalis, the posterior, often dorsal side, of the sterigma. The section of the ductus bursae near the ostium bursae is called the antrum, and a sclerotized structure just below it, or anterior to it, if present, is called a colliculum.

## Results

The adapted key below using external and tympanal characters from Munroe and Solis (1998) can aid in identifying a species as Crambinae.

### Key to Crambinae in Relation to the other Subfamilies of Pyraloidea

(adapted from Munroe and Solis 1998)

**Table:** 

1	Praecinctorium absent; tympanal case “closed” medially and open anteriorly only; tympanum and conjunctiva in the same plane	**Pyralidae**
–	Praecinctorium present; tympanal case “open” anteromedially; tympanum meeting conjunctiva at a distinct angle	**Crambidae, 2**
2	Chaetosema absent; forewing with distal part of CuP developed as a tubular vein; proboscis present, but reduced; tympanal cases reduced and widely separated; praecinctorium reduced	other **Crambidae**
–	Chaetosema present; forewing with CuP absent, not developed as a tubular vein; proboscis usually present and tympanal organs almost always normally developed	**3**
3	R_2_ of forewing at least closely apposed to and usually stalked with R_3+4_; labial palpus usually upturned, basal segment often longer than second segment; wings mostly with conspicuous pattern of transverse bands on a pale ground; larvae aquatic, rarely in damp terrestrial habitats	other **Crambidae**
–	R_2_ of forewing well separated from R_3+4_; labial palpus often porrect, basal segment much shorter than second segment; wings usually without conspicuous pattern of transverse bands on a pale ground; larvae usually terrestrial, sometimes stem borers in aquatic graminaceous plants	**4**
4	Forewing usually with weakly raised patches of black scales; cubitus of hindwing usually not pectinated with hairlike scales; lateral arms of tegumen of male genitalia about as long as uncus, little tapered ventrally; uncus of moderate length, pyriform, hoodshaped or bilobed, not obviously decurved from base to tip; gnathos with median element spikelike, sword-shaped, or digitate, straight or decurved, rarely reduced; uncus and gnathos not forming a jawlike structure, and not widely separated dorsad from valvae; valva sometimes with a ventral process but, except in *Heliothela*, without strong costal or medial armature; known larvae on mosses, lycopods, ferns, and roots of seed-bearing vascular plants	**Scopariinae**
–	Forewing without raised patches of black scales; cubitus of hindwing usually pectinated with hairlike scales, lateral arms of tegumen of male genitalia much longer than uncus or narrowed ventrally, usually both; uncus usually long, acuminate, and more or less decurved from base to tip; gnathos with lateral arms articulating at base of uncus, medial element various in shape, often rodlike or forming a dorsally directed hook; uncus and gnathos forming a jawlike structure, widely separated dorsad from valvae; valva often with strong costal process or medial armature; larvae mostly feeding at bases, roots, stems of grasses (Poaceae)	**Crambinae**

### Diagnosis of *Diatraea*

In the Crambinae *Diatraea* is morphologically defined by a combination of derived characters including a lack of ocelli on the head (absent or reduced in the externally similar *Donacoscaptes* and *Xubida* (B. Landry, pers. comm.)), the presence of pockets with specialized scales on the male second abdominal segment, hair tufts on the male hind tibia, in the male genitalia basal extensions of the tegumen in some or most species (Landry 1995). Landry (1995) also suggested that the shape of the female sterigma with shallow sclerotized, often spinose, depressions on each side of the ostium bursae, may be unique to *Diatraea* (in contrast to the externally similar *Donacoscaptes* and *Xubida* where “the setation of the female segment VIII is concentrated apico-dorsally” and “the female sterigma and segment VIII are sometimes linked by a narrow sclerotized bridge which may be single or double” (Landry 1995)). Another potentially derived structure in the male genitalia could be the lack of muscle attachments in the lateral lobes of the tegumen (Solis and Metz 2011).

### Checklist of *Diatraea* Guilding, 1828

***Diatraea* Guilding, 1828**: 148. Type species: *Diatraea sacchari* Guilding, 1828: 149 by monotypy.

*Diatrea* Guilding, 1828: plate 12. Misspelling.

*Diaraetria* Grote, 1882: 56. Misspelling.

*Iesta* Dyar, 1909: 29. Type species: *Iesta lisetta* Dyar, 1909: 29 by original designation.

*Diatraerupa* Schaus, 1913: 240. Type species: *Diatraerupa guapilella* Schaus, 1913: 240 by original designation.

*Trinidadia* Dyar & Heinrich, 1927: 5. Type species: *Diatraea minimifacta* Dyar, 1911: 202 by original designation.

*Eodiatraea* Box, 1953: 178. Type species: *Chilo centrellus* Möschler, 1883: 360 by original designation.

*Crambidiatraea* Box & Capps, 1955: 175. Type species: *Diatraea cayennella* Dyar & Heinrich, 1927: 27 by original designation.

*Zeadiatraea* Box, 1955: 197. Type species: *Leucania lineolata* Walker, 1856: 100 by original designation.

***Diatraea albicrinella* Box, 1931**: 34. Type locality: Fonte Boa, Amazonas, Brazil.

***Diatraea andina* Box, 1951**: 393. Type locality: near Cordero, Upper Rio Torbes, Tachira, Venezuela.

***Diatraea argentina* Box, 1931**: 18. Type locality: nr. Florenzia, Gran Chaco, Argentina.

***Diatraea bellifactella* Dyar, 1911**: 205. Type localities: Sáo [São] Paulo, Brazil (male) and Castro, Parana, Brazil (female).

*Diatraea balboana* Box, 1956: 769. Type locality: Summit Botanical Gardens, Panama City, Panama. **Syn. n.**

***Diatraea brunnescens* Box, 1931**: 29. Type locality: Ciudad Bolivar, Venezuela.

*Diatraea incertella* Box, 1931: 30. Type locality: Rio de Janeiro, Brazil.

***Diatraea busckella* Dyar & Heinrich, 1927**: 16. Type locality: Porto Bello, Panama.

*Diatraea luteella* Box, 1931: 32. Type locality: Rio Cayapas, Esmeraldas, Ecuador. **Syn. n.**

*Diatraea rosa* Heinrich, 1931: 4. Type locality: Carabobo, Venezuela. **Syn. n.**

*Diatraea busckella forma falconensis* Box, 1951: 389. Type localities: Piritú and Cumarebo, Falcon, and Ocumare de la Costa, Aragua,Venezuela. **Syn. n.**

*Diatraea busckella setariae* Box, 1951: 391. Type locality: near Yuma promontory, Carabobo, Venezuela. **Syn. n.**

*Diatraea setariaeoides* Box, 1951: 390. Type locality: Ocumare de la Costa, Aragua, Venezuela. **Syn. n.**

*Diatraea colombiana* Box, 1956: 768. Type locality: Condoto, Prov. Choco, Colombia. **Syn. n.**

***Diatraea castrensis* Dyar & Heinrich, 1927**: 28. Type locality: Castro, Parana, Brazil.

***Diatraea cayennella* Dyar & Heinrich, 1927**: 27. Type locality: Cayenne, Fr. Guiana.

*Diatraea cayenella* Box, 1931: 38. Misspelling.

***Diatraea centrella* (Möschler, 1883)**: 360 (*Chilo*). Type locality: Paramaribo, Surinam.

*Phalaena sacchari* Sepp, 1848: 135. Type locality: Surinam.

*Diatraea canella* Hampson, 1895: 349. Type localities: Balthazar, Grenada or Mount Gay Estate, [Barbados], or Brazil.

*Diatraea amnemonella* Dyar, 1911: 203. Type locality: Castro, Parana, Brazil. **Syn. n.**

*Diatraea anathericola* Dyar & Heinrich, 1927: 21. Type locality: Sāo Paulo, Brazil.

*Diatraea amazonica* Box, 1931: 36. Type locality: Calama, R. Madeira, Brazil. **Syn. n.**

***Diatraea considerata* Heinrich, 1931**: 3. Type locality: Eldorado, Sinaloa, Mexico.

***Diatraea crambidoides* (Grote, 1880)**: 50 (*Chilo*). Type locality: Kansas, USA.

*Diatraea zeacolella* Dyar, 1911: 203. Type locality: Tryon (Polk County), North Carolina, USA.

*Diatraea tripsacicola* Dyar, 1921: 193. Type locality: Miami, Florida, USA.

***Diatraea dyari* Box, 1930**: 307. Type locality: San Pedro de Jujuy, Jujuy, Argentina.

***Diatraea evanescens* Dyar, 1917**: 84. Type locality: Audubon Park, Louisiana, USA.

*Diatraea sobrinalis* Schaus, 1922: 140. Type locality: Cayuga, Izabal, Guatemala.

***Diatraea fuscella* Schaus, 1922**: 139. Type locality: Carillo, Costa Rica.

***Diatraea gaga* Dyar, 1914**: 319. Type locality: Corozal, Canal Zone, Panama.

*Diatraea solipsa* Dyar, 1914: 319. Type locality: Porto Bello, Colón, Panama, or Trinidad River, Panama.

*Diatraea savannarum* Box, 1935: 332. Type locality: Rupununi savannahs, base of Shiriri Mt., Guyana. **Syn. n.**

***Diatraea grandiosella* Dyar, 1911**: 205. Type locality: Guadalajara, Mexico.

***Diatraea guatemalella* Schaus, 1922**: 138. Type locality: Cayuga, Guatemala.

***Diatraea impersonatella* (Walker, 1863)**: 163 (*Crambus*). Type locality: Venezuela.

Note: Described from a series of 7 specimens from Venezuela and Brazil. Box (1931: Plate III) figured two female genitalia dissections (BMNH #141 and #142) that we studied. Figured in this paper is Figure 26d that is BMNH #142 (= Box #3). Box considered the dissected female, BMNH #141, to be the last remaining syntype of Walker’s original series. The locality is unknown, but presumed by Box (1931:41-42) to be Venezuela: “The only specimen which we can to-day assert to have been included among the above, is the female type in the British Museum from Venezuela.”

Note: This appears to be a variable species based on the number of specimens available and barring numerous dissections. Despite differences in size and coloration, the male genitalia are consistent throughout with the same morphology for the uncus and gnathos, lateral process of the tegumen, and costal processes. The females, however, are not consistent. In the *impersonatella* form, the lamella postvaginalis has the transverse ridges at an angle so that medially they are farther from the ostium bursae than the lateral ends and the membranous area in the middle is wide and widens past the ridges forming an hourglass shape. In the *moorella* form it is either like the *impersonatella* form or has the transverse ridges completely absent and the lamella postvaginalis a large roughened patch without a wide membranous area in the middle. The *pallidostricta* and *flavipennella* (and some *moorella*) form females have a much rounder lamella postvaginalis that is often glabrous and the transverse ridges are arcuate. Nomenclaturally, the synonomy for this group of names is also confounded by the lack of single typification for the syntype series and that some of these species are represented by female holotypes among what seems to be variable female genitalia.

*Diatraea angustella* Dyar, 1911: 205. Type locality: Castro, Parana, Brazil. **Syn. n.**

*Diatraea angustellus* Dyar, 1911. Schaus, 1922: 140. Misspelling.

*Diatraea moorella* Dyar & Heinrich, 1927: 17. Type locality: Georgetown, Guyana.

*Diatraea flavipennella* Box, 1931: 42. Type locality: Castro, Parana, Brazil. **Syn. n.**

*Diatraea pallidostricta* Dyar, 1911: 205. Type locality: São Paulo, Brazil. **Syn. n.** Note: Bleszynski (1967) considered *Diatraea pallidostricta* a junior synonym of *Zeadiatraea lineolata* in the World Catalog, but this was a mistake; it was repeated in Munroe et al. (1995).

***Diatraea indigenella* Dyar & Heinrich, 1927**: 13. Type locality: Popayán, Colombia.

***Diatraea instructella* Dyar, 1911**: 201. Type locality: Popocatepetl Park, Mexico.

Note: Only known from two specimens, the female holotype in the USNM and a male that Box determined to be conspecific based on external characters and type locality. The male and female may not actually be conspecific.

***Diatraea lativittalis* (Dognin, 1910)**: 117 (*Chilo*). Type locality: Tucuman, Argentina.

*Diatraea obliqualis* Hampson, 1919: 543. Type locality: Goya, Corrientes, Argentina. **Syn. n.**

*Chilo latmiadelis* Dognin, 1923: 38. Unjustified emendation/replacement of *lativittalis* (Dognin, 1910) (nec. *lativittalis* (Walker, 1863: 171) (*Chilo*)).

***Diatraea lentistrialis* Hampson, 1919**: 546. Type locality: Florenzia, Gran Chaco, Argentina.

*Diatraea silvicola* Box, 1951: 396. Type locality: Guasdualito El Amparo road, Upper Apure, Venezuela. **Syn. n.**

***Diatraea lineolata* (Walker, 1856)**: 100 (*Leucania*). Type locality: Venezuela.

*Chilo culmicolellus* Zeller, 1863: 7. Type locality: Colombia.

*Chilo neuricellus* Zeller, 1863: 8. Type locality: Venezuela.

***Diatraea lisetta* (Dyar, 1909)**: 29 (*Iesta*). Type locality: Dade City, Florida, USA.

*Iesta cancellalis* Dyar, 1914: 320. Type locality: Corozal, Canal Zone, Panama.

*Iesta adulcia* Dyar, 1916: 37. Type locality: Teapa, Tabasco, Mexico.

***Diatraea magnifactella* Dyar, 1911**: 201. Type localities: Orizaba, Cuernavaca, and Oaxaca, Mexico.

***Diatraea maronialis* Schaus, 1922**: 139. Type locality: St. Jean, Maroni River, French Guiana.

*Diatraea umbrialis* Schaus, 1922: 139. Type locality: St. Jean, Maroni River, French Guiana. **Syn. n.**

***Diatraea minimifacta* Dyar, 1911**: 202. Type locality: Trinidad, B.W. I.

*Diatraerupa guapilella* Schaus, 1913: 240. Type locality: Guápiles, Limón, Costa Rica. **Syn. n.**

*Iesta morobe* Dyar, 1916: 37. Type locality: Teapa, Tabasco, Mexico. **Syn. n.**

*Diatraea pittieri* Box, 1951: 394. Type locality: Rancho Grande, Aragua, Venezuela. **Syn. n.**

***Diatraea mitteri* Solis, 2015**: 649. Type locality: Woodward, Oklahoma, United States.

***Diatraea muellerella* Dyar & Heinrich, 1927**: 25. Type locality: Guerrero, Mexico.

***Diatraea myersi* Box, 1935**: 331. Type locality: Recreio, Amazons, Brazil.

***Diatraea pedibarbata* Dyar, 1911**: 202. Type locality: St. Laurent du Maroni, French Guiana.

*Diatraea maritima* Box, 1935: 333. Type localities: Plantation Ogle, Plantation Albion and Georgetown, Guyana. **Syn. n.**

***Diatraea postlineella* Schaus, 1922**: 138. Type locality: Quirigua, Guatemala.

***Diatraea ragonoti* Box, 1948**: 421. Type locality: Petropolis, Rio de Janeiro, Brazil.

***Diatraea rufescens* Box, 1931**: 37. Type locality: Buenavista, Bolivia.

***Diatraea saccharalis* (Fabricius, 1794)**: 238, 411 (*Phalaena*). Type locality: America Meridionalis [presumably Surinam (Box, 1931: 23)].

*Crambus sacchari* Fabricius, 1798: 469, 31. Unjustified emendation of *saccharalis* Fabricius, 1794: 469.

*Diatraea sacchari* Guilding, 1828: 149. Junior homonym of *sacchari* Fabricius, 1798: 469.

*Crambus leucaniellus* Walker, 1863: 161. Type locality: St. Domingo, West Indies.

*Crambus lineosellus* Walker, 1863: 162. Type locality: Honduras.

*Chilo obliteratella* Zeller, 1863: 8. Type locality: Brazil.

*Diatraea grenadensis* Dyar, 1911: 200. Type locality: Grenada, West Indies.

*Diatraea pedidocta* Dyar, 1911: 201. Type locality: Cordoba, Mexico.

*Diatraea continens* Dyar, 1911: 202. Type locality: Castro, Parana, Brazil.

*Diatraea brasiliensis* van Gorkum & de Waal, 1913: 181. Type locality: Brazil.

*Diatraea incomparella* Dyar & Heinrich, 1927: 13. Type locality: Taperinha, Para [Amazonas], Brazil.

*Diatraea centinens* Dyar & Heinrich, 1927: 7. Misspelling.

***Diatraea schausella* Dyar & Heinrich, 1927**: 24. Type locality: Chejel, Alta Verapaz, Guatemala.

Note: Known only from male specimens. The single female specimen noted by Box (1931:45) and labeled “COLOMBIA, Choko Prov., Condoto (H. G. F. Spurrell)” is likely *Diatraea busckella* Dyar & Heinrich, 1927.

***Diatraea strigipennella* Dyar, 1911**: 206. Type localities: “Guianas” and Castro, Parana, Brazil.

*Diatraea entreriana* Box, 1931: 39. Type locality: La Soledad, Entre Rios, Argentina. **Syn. n.**

***Diatraea suffusella* Box, 1931**: 33. Type locality: St. Jean du Maroni, French Guiana.

***Diatraea tabernella* Dyar, 1911**: 200. Type locality: Tabernilla, Panama Canal Zone, Panama.

***Diatraea venosalis* (Dyar, 1917)**: 87 (*Haimbachia*). Type locality: Audubon Park, Louisiana, USA.

***Diatraea veracruzana* Box, 1956**: 770. Type locality: Teocelo, near Coatepec, Veracruz, Mexico.

### Key to the species of *Diatraea* based on male genitalia

[*Diatraea lativittalis* (Dognin, 1910) and *Diatraea suffusella* Box, 1931 are known from only female specimens, so they cannot be identified with this key.]

**Table:** 

1	Uncus broad at apex, paddle-shaped (*bellifactella* group) (Figs 8d; 9a, c)	**2**
1’	Uncus triangular, narrowing at apex, beaklike	**4**
2 (1)	Tegumen with crenulate, lateral lobes; uncus stiff, extended ventrolaterally, but not bilobed (Fig. 8d)	***fuscella***
2’	Tegumen at most carinate laterally, lacking lobes; uncus less sclerotized and bilobed (Figs 9a, c)	**3**
3 (2’)	Juxta with four lateral projections, two central, long, and two lateral, short (Fig. 9d)	***andina***
3’	Juxta with three projections, two lateral, long, and one central, shorter, (Fig. 9a)	***bellifactella***
4 (1’)	Juxta with three projections, two lateral and one central (*strigipennella* group) (Figs 7b,d; 8b)	**5**
4’	Juxta with two lateral projections	**7**
5 (4)	Central projection of juxta more than five times longer than wide (Fig. 7b)	***strigipennella***
5’	Central projection of juxta less than three times longer than wide (Figs 7d, 8b)	**6**
6 (5)	Medial portion of basal costal lobe on valva crenulate almost as broad as long (Fig. 8b)	***cayennella***
6’	Medial portion of basal costal lobe on valva smooth, slender, acutely pointed (Fig. 7d)	***castrensis***
7 (4’)	Valva costal margin with narrow, accessory process (Fig. 10b, e, red arrow); basal costal lobe present or absent (*centrella* group)	**8**
7’	Valva costal margin lacking narrow accessory process, basal costal lobe present or absent	**9**
8 (7)	Valva accessory process on costal margin curved, face of juxta arms with denticles (Fig. 10e)	***rufescens***
8’	Valva accessory process on costal margin straight, juxta arms with denticles only on posterior edge (Fig. 10b)	***centrella***
9 (7’)	Apex of juxta arms bidentate, with two distinct points (*lineolata* group) (Figs 11b,e, red arrow; 12b, e)	**10**
9’	Apex of juxta arms with a single point or rounded with a small, subapical tooth, but never bidentate	**13**
10 (9)	Apex of juxta arms cylindrical, apical teeth subequal in size, clawlike (Fig. 11b)	***lineolata***
10’	Apex of juxta arms flat, apex pointed with smaller subapical tooth (Figs 11e, 12b, e)	**11**
11 (10’)	Apex of lateral juxta arms spatulate, gnathos with large, pointed process in middle (Fig. 11d)	***muellerella***
11’	Apex of lateral juxta arms attenuate, gnathos without process (Fig. 12b, e)	**12**
12 (12’)	Apex of gnathos bluntly rounded and denticulate, apex of uncus pointed (Fig. 12d)	***schausella***
12’	Apex of gnathos and uncus spatulate (Fig. 12a)	***grandiosella***
13 (9’)	Teeth on gnathos 6× longer than wide or longer, like setae (Figs 12g, j; 19d) (*crambidoides* group)	**14**
13’	Teeth on gnathos no more than 4×longer than wide, short like serrations on a butter knife, or completely absent	**16**
14 (13)	Uncus drastically narrowing before apex, then apex slightly capitate and cleft, lateral edges of uncus rough; gnathos recurving back on itself noticeably (Fig. 19d)	.***mitteri***
14’	Uncus slightly narrowing towards apex, but not noticeably capitate, apex rounded or pointed, lateral edges smooth or carinate; gnathos slightly hooked, but not recurving back towards base (Fig. 12g, j)	**15**
15 (14’)	Ventrolateral edges of uncus medially expanded into wide blades, apex broadly spatulate (Fig. 12g)	***crambidoides***
15’	Ventrolateral edges of uncus medially carinate, but not expanded, apex flat, but not broadly spatulate (Fig. 12j)	***postlineella***
16 (13’)	Lateral edge of tegumen without lobelike process (lateral lobes of D. & H., 1927) (*lisetta* group) (Figs 2a, d; 4d; 5c)	**17**
16’	Lateral edge of tegumen with lobelike process, sometimes small and hard to see (as in *gaga*) (Fig. 3d)	**25**
17 (16)	Juxta constricted laterally before base of juxta arms, juxta arms emerging more medially (Fig. 5d)	***minimifacta***
17’	Juxta evenly rounded to base of juxta arms, juxta arms emerging more laterally	**18**
18 (17’)	Valva costal margin significantly extended posteriorly, apically of basal costal lobe, basal costal lobe present (Figs 2b, 4e)	**19**
18’	Valva costal margin not significantly extended posteriorly, apically of basal costal lobe, basal costal lobe absent or present (Fig. 4b)	**20**
19 (18)	Apex of basal costal lobe of valva sharply pointed (Fig. 2b)	***argentina***
19’	Apex of basal costal lobe of valva evenly rounded (Fig. 4e)	***lisetta***
20 (18’)	Apex of juxta arms rounded with subapical tooth (Fig. 4b)	***lentistrialis***
20’	Apex of juxta arms pointed (Figs 2e, 3e, 6g)	**21**
21 (20’)	Gnathos thin and cylindrical (Fig. 2d)	***brunnescens***
21’	Gnathos flattened and beaklike (Fig. 6e)	**22**
22 (21’)	Gnathos with a pronounced mound of teeth subapically (Fig. 6e, f)	***venosalis***
22’	Gnathos with teeth, but not on a distinct, subapical mound	**23**
23 (22’)	Valva without basal costal extension (Fig. 3e)	***gaga*** in part
23’	Valva with bluntly pointed basal costal extension, but no real lobe (Figs 5b, 6c)	**24**
24 (23’)	Gnathos in lateral view more-or-less straight from base to tip, dorsal surface slightly undulate, but not arcuate, only tip with slight hook; tegumen in lateral view larger at the base than at the point of articulation with uncus/gnathos; brush of setae at tip of valva dense and long, length more than twice width of valva where brush emerges from valva (Fig. 5a, b)…	***maronialis***
24’	Gnathos in lateral view arcuate, middle of dorsal surface “lower” than base and tip; tegumen in lateral view more-or-less equal in width throughout length; brush of setae at tip of valva only slightly more conspicuous than rest of setae on valva, length subequal to width of valva where brush emerges from valva (Fig. 6a, b)	***myersi***
25 (16’)	Small species, juxta arms acutely pointed apically with no subapical tooth (Fig. 3b, e)	**26**
25’	Large species, juxta arms rounded apically, subapical tooth absent or present (*saccharalis* group) (Fig. 13b, e)	**27**
26 (25’)	Lateral lobes of tegumen conspicuous, at least as long as wide at base (Fig. 3a)	***evanescens***
26’	Lateral lobes of tegumen obscure, no more than half as long as wide at base (Fig. 3d)	***gaga*** in part
27 (25’)	Lateral lobe of tegumen square, either with corners sharp or rounded (Figs 16d, 17d)	**28**
27’	Lateral lobe of tegumen rounded or pointed	**29**
28 (27)	Lateral lobe of tegumen square with rounded corners (Fig. 16d)	***magnifactella***
28’	Lateral lobe of tegumen square with sharp corners (Fig. 17d)	***ragonoti***
29 (27’)	Lateral lobe of tegumen broadly rounded, basal width 2/3 length of tegumen (Fig. 13a)	***albicrinella***
29’	Lateral lobe of tegumen less broad, basal width at most 1/2 length of tegument	**30**
30 (29’)	Valva basal costal lobe triangular, not significantly produced, lacking crenulations, carinae, or denticulation, setose only (Fig. 14e)	***dyari***
30’	Valva basal costal lobe globular, at least partially, significantly produced, with at least some crenulation, carinae, or denticulation	**31**
31 (30’)	Valva basal costal lobe weakly crenulate dorsally (Fig. 17b)	***pedibarbata***
31’	Valva basal costal lobe strongly crenulate, carinate and/or denticulate	**32**
32 (31’)	Lateral lobe of tegumen sharply pointed, apex flattened anteroposteriorly (Figs 14g, 16a)	**33**
32’	Lateral lobe of tegumen bluntly pointed or rounded	**34**
33 (32)	Uncus with ventrolateral, carinate margin constricting sharply and is not carinate just before apex of uncus, thus making apex slightly spatulate; basal costal lobe of valva capitate, posterior surface evenly rounded and lacking a depression; transition between basal costal lobe and following section of costa smooth, not notched (Fig. 16a, b)	***indiginella***
33’	Uncus with a ventrolateral, carinate margin that is complete, not tapered before reaching apex; basal costal lobe of valva protruding posteriorly only, not widened laterally at apex, with small depression in posterior surface; with roughened notch between base of basal costal lobe and following section of costa (Fig. 14g, h)	***instructella***
34 (32’)	Valva basal costal lobe base with basal and apical widths subequal, entire lobe wider than long (Fig. 14b)	***considerata***
34’	Valva basal costal lobe narrower at base than apex, essentially capitate (Figs 18e, 19b) (subtle or not clearly evident in *Diatraea saccharalis*)	**35**
35 (34’)	Lateral lobe of tegumen clearly ovate (Fig. 18a)	**36**
35’	Lateral lobe of tegumen bluntly pointed (Figs 15a, 19a)	**37**
36 (35)	Lateral lobe of tegumen as long as wide (Fig. 18a)	***saccharalis***
36’	Lateral lobe of tegumen longer than wide (Fig. 18d)	***tabernella***
37 (35’)	Anterior edge of lateral lobe of tegumen angled, perpendicular to tegumen at base, then turning posteroventrally (Fig. 19a)	***veracruzana***
37’	Anterior edge of lateral lobe of tegumen straight or arcuate, but not angled	**38**
38 (37’)	Anterior edge of lateral lobe of tegumen straight, denticulation on valva basal costal lobe large and densely packed on lobe (Fig. 15a, b)	***guatemalella***
38’	Anterior edge of lateral lobe of tegumen arcuate, denticulation on valva basal costal lobe large or small, but not densely packed on lobe (Figs 13d, e; 15d, e)	**39**
39 (38’)	Denticulation of valva basal costal lobe small, lobe essentially not darkened more than rest of cuticle as a result of denticulation (Fig. 15e)	***impersonatella***
39’	Denticulation of valva basal costal lobe large, lobe darkened more than rest of cuticle as a result of denticulation (Fig. 13e)	***busckella***

### Simple key to *Diatraea* species based on female genitalia

[The female is unknown for the following species and therefore not included in the key below: *castrensis* Dyar & Heinrich, 1927; *schausella* Dyar & Heinrich, 1927. We did not have female specimens on hand of *ragonoti* Box, 1948 and *suffusella* Box, 1931.]

**Table:** 

1	Corpus bursae with darkly sclerotized, teethlike spines or flattened plates (Figs 21c, 22d)	**2**
1’	Corpus bursae completely membranous, at most with areas of darkened cuticle	**3**
2 (1)	Lamella antevaginalis hardened and darkened, appearing as a medially-bisected plate that protrudes posteriorly over the genital opening; corpus bursae with a ring of sclerotized flattened plates (Fig. 22d)	***strigipennella***
2’	Lamella antevaginalis membranous, possibly sclerotized as much as sternites, but not dark or protruding over the genital opening; corpus bursae with opposite patches of sclerotized teethlike spines (Fig. 21c)	***lentistrialis***
3 (1’)	Sternite VIII with a broad, transverse “pocket” mostly concealing ostium bursae; lamella antevaginalis composed of a pair of hardened, posteriorly projecting extensions that may cover the genital opening or surround it laterally; lamella postvaginalis with lateral areas of wrinkled and/or densely setose cuticle contrasting strikingly with medial area that is smooth and glabrous; or if lamella postvaginalis immediately posterad ostium bursae smooth and concave then with a pair of densely setose transverse ridges posterad concavity that project ventrally (*saccharalis* group) (Fig. 28a)	**4**
3’	Sternite VIII with ventral surface continuous with ostium and membranous never forming a transverse pocket concealing ostium; or if lamella antevaginalis and/or lamella postvaginalis varously sclerotized and ostium concealed then without contrasting lateral areas of roughened or densely setose cuticle and a pair of densely setose transverse ridges posterad	**12**
4 (3)	Corpus bursae more than 5× longer than wide; corpus bursae shape cylindrical, more or less parallel sided (Fig. 26c)	***guatemalella***
4’	Length of corpus bursae variable, but if longer than wide then less than 5× longer than wide; corpus bursae shape variable, but usually irregular to ovate	**5**
5 (4’)	Lateral, wrinkled and densely setose cuticle of lamella postvaginalis continuous with and continuing laterally along posterior margin of sternite VIII, not forming subcircular patches (Fig. 27b)….	***instructella***
5’	Lateral, wrinkled and/or densely setose cuticle of lamella postvaginalis not reaching posterior margin of sternite VIII, or if approximate to posterior margin forming distinct subcircular patches contrasting with the rest of sternite VIII cuticle (Fig. 27d)	**6**
6 (5’)	Lamella postvaginalis lacking a distinct pair of transverse ridges posterad, cuticle wrinkled or densely setose, but solitary set of transverse ridges undetectable (Figs 26a; 27a, c, d)	**7**
6’	Lamella postvaginalis with a distinct pair of transverse ridges posterad, ridges distinct from surrounding cuticle	**9**
7 (6)	Corpus bursae only slightly longer than wide, not extending or barely extending beyond anterior margin of sternite VIII (Fig. 27d)	***pedibarbata***
7’	Corpus bursae length at least 2X greater than width, extending well beyond anterior margin of sternite VIII	**8**
8 (7’)	Ductus bursae with longitudinal grooves, uniformly darkened throughout length from ostium to corpus bursae; corpus bursae with pair of acuminate strips of darker cuticle descending from ductus bursae (Figs 26a, 27c)	***considerata* or *magnifactella***
8’	Ductus bursae more or less smooth, lightly darkened except for contrasting colliculum at junction with corpus bursae that is considerably darker; corpus bursae without any darkened areas at base, completely membranous (Fig. 27a)	***indigenella***
9 (6’)	Corpus bursae length 4× greater than width, long and narrow with lateral expansions near middle no wider than width of corpus; posteriorly projecting extensions of lamella antevaginalis reduced, mostly membranous (Fig. 28c)	***veracruzana***
9’	Corpus bursae length 2× greater than width or less, irregularly shaped or ovate, width at middle appearing to contribute to overall shape of corpus rather than as lateral expansions; posteriorly projecting extensions of lamella antevaginalis enlarged and conspicuous, middle membranous area much narrower	**10**
10 (9’)	Densely setose, ventrally projecting transverse ridges encompassing posterior half of lamella postvaginalis, flat anterad only, forming broad pillow shapes in posterior half of lamella postvaginalis (Fig. 25f)	***busckella***
10’	Densely setose, ventrally projecting transverse ridges forming a narrow band in lamella postvaginalis, flat both anterad and posterad, forming an elevated crest at posterior margin of lamella postvaginalis (Fig. 28b)	**11**
11 (10’)	Posterior margin of hardened, posteriorly projecting extensions of lamella antevaginalis smooth and broadly arcuate, forming an almost semicircular arc from medial to lateral edge (Fig. 28b)….	***tabernella***
11’	Posterior margin of hardened, posteriorly projecting extensions of lamella antevaginalis irregularly shaped with at least one substantial notch or indentation, bluntly pointed, forming a triangle (Figs 25e, 26d, 28a)	***albicrinella*, *impersonatella*, or *saccharalis***
12 (3’)	Lamella antevaginalis and/or lamella postvaginalis variously sclerotized and adorned with texture markedly different than remaining cuticle of sternite VIII (Figs 23a,c,d)	**13**
12’	Lamella antevaginalis and/or lamella postvaginalis unadorned, cuticle around ostium not significantly dissimilar to cuticle of rest of sternite VIII	**15**
13 (12)	Lamella antevaginalis with rugose cuticle continuous from sternite VIII to hardened, posteriorly projecting, cylindrical extensions around rim of ostium bursae that are bisected medially by a hardened slot (Fig. 23a)	***cayennella***
13’	Lamella antevaginalis with a raised ring of semicircular cuticle separating sternite VIII cuticle from margin of ostium bursae by a semimembranous depression	**14**
14 (13’)	Raised circular cuticle of lamella antevaginalis darkened around entire edge, medially as dark as laterally; lamella antevaginalis laterally with denser/larger setal sockets, but not rugose; rim of ostium bursae and beginning of antrum irregularly notched and grooved; lamella postvaginalis smooth medially, membranous (Fig. 23d)	***andina***
14’	Raised circular cuticle of lamella antevaginalis not darkened medially, noticeably darker on lateral edges; lamella antevaginalis laterally with rugose cuticle in addition to more densely spaced setal sockets; rim of ostium bursae with a single medial notch; lamella postvaginalis medially with semicircular ridges resembling a thumbprint (Fig. 23c)	***bellifactella***
15 (12’)	Antrum with heavy sclerotization in the shape of a yoke, narrower at dorsal margin of ostium bursae, widening laterally and descending down antrum, lateral edges folded inward forming a trough (Fig. 20b)	***brunnescens***
15’	Antrum variously sclerotized, but sclerotization not shaped like a yoke or lateral edges folded into a trough	**16**
16 (15’)	Antrum and ductus bursae only lightly sclerotized or not at all, colliculum not evident; union of ductus bursae and corpus bursae smooth, not constricted, so that beginning of corpus is indistinct (Fig. 26b)	***dyari***
16’	Antrum and ductus bursae with some sclerotization and colliculum usually present; if sclerotization of membrane light or indistinct then terminus of ductus bursae and beginning of corpus bursae always obvious	**17**
17 (16’)	Terminal end of ductus bursae with a spherical, membranous expansion that is 2× wider than ductus bursae before pinching to opening of corpus bursae (Fig. 22b)	***myersi***
17’	Ductus bursae with varying shapes and widths, but never with a large, spherical expansion before the opening of the corpus bursae	**18**
18 (17’)	Corpus bursae medially wider due to presence of shallow lateral pockets on each side about midway, not simply oval-shaped	**19**
18’	Corpus bursae oval shaped, without shallow lateral pockets on each side	**20**
19 (18)	Ostium bursae and antrum equal in width, but ductus bursae narrower forming a constriction before corpus bursae (Fig. 20c)	***evanescens***
19’	Ostium bursae, antrum, and ductus bursae subequal in width, constriction if any before corpus bursae subtle (Fig. 20d)	***gaga***
20 (18’)	Lamella postvaginalis with long setae near ostium bursae (Fig. 21a)	***lativittalis***
20’	Lamella postvaginalis without long setae near ostium bursae	**21**
21 (20’)	Corpus bursae length at least 4× width, long and narrow, more cylindrical than oval	**22**
21’	Corpus bursae length no more than 3× width, more oval or irregularly shaped	**23**
22 (21)	Central America north to southeast United States (Fig. 21d)	***lisetta***
22’	Argentina (Fig. 20a)	***argentina***
23 (21’)	Margin of ostium bursae, antrum, AND ductus bursae without wrinkles, ridges, grooves, or undulations (Fig. 23b)	***fuscella***
23’	At least some part of margin of ostium bursae, antrum, OR ductus bursae with wrinkles, ridges, grooves, and/or undulations	**24**
24 (23’)	Colliculum a broad band notched in the middle to form a saddle shape; distinctly demarcated and darker than rest of surrounding cuticle	**25**
24’	Colliculum variable or indistinct, but if distinct from surrounding cuticle, not a broad, saddle-shaped band notched in the middle	**26**
25 (24)	Ostium bursae circular, margin less undulate, only slightly uneven; ductus bursae length at least 2× width, with longitudinal ridges (Fig. 22c)	***venosalis***
25’	Ostium bursae flattened dorsoventrally, ventral margin distinctly undulate; ductus bursae length subequal to width or only slightly longer, lacking longitudinal ridges (Fig. 24d)	***muellerella***
26 (24’)	Papillae analis with lobelike ventral extension, distinct from smooth, outer sweep of papillae analis (Fig. 25a, b)	***grandiosella***
26’	Papillae analis normally rounded ventrally, outer sweep continuous (Fig. 25c, d)	**27**
27 (26’)	Ostium bursae completely open to environment, not concealed by any surrounding cuticle of the lamella antevaginalis or lamella postvaginalis other than undulations of margin	**28**
27’	Ostium bursae partially enclosed by pinching of lamella antevaginalis or lamella postvaginalis, opening to environment narrower than ostium bursae (Figs 22a, 24a, b)	**31**
28 (27)	Distance between ostium bursae and posterior margin of sternite VIII subequal to width of ostium bursae; ductus bursae length nearly 2× width, noticeably more darkly sclerotized ventrally (Fig. 21e)	***maronialis***
28’	Distance between ostium bursae and posterior margin of sternite VIII at least 2× width of ostium bursae; ductus bursae length subequal to width, ventrally no darker than remaining membrane	**29**
29 (28’)	Ventral and lateral margins of ostium bursae distinctly undulate with deep invaginations forming ridges in beginning of antrum (Fig. 24c)	***lineolata***
29’	Ventral and lateral margins of ostium bursae barely roughened, walls of antrum unaffected by shape of margin of ostium bursae (Fig. 25c)	**30**
30 (29’)	Sclerotized collar on ductus bursae triangular, surface denticulate, edges jagged, midlength shorter than width of ductus bursae (Fig. 25c)	***crambidoides***
30’	Sclerotized collar on ductus bursae rectangular, surface and edges smooth, midlength subequal to width of ductus bursae (Fig. 29b)	***mitteri***
31 (27’)	Lamella antevaginalis expanded posteriorly over ostium bursae, split in the middle forming a notch between cuticle and exposing ostium bursae; lamella postvaginalis widened into circular opening with wrinkled edges (Fig. 22a)	***minimifacta***
31’	Lamella antevaginalis only slightly extending over ostium bursae with wrinkled ventral margin and without a noticable notch; lamella postvaginalis pinched medially forming a notch over ostium bursae and a groove posterad (Fig. 24a, b)	***centrella* or *rufescens***

**Figure 3.**
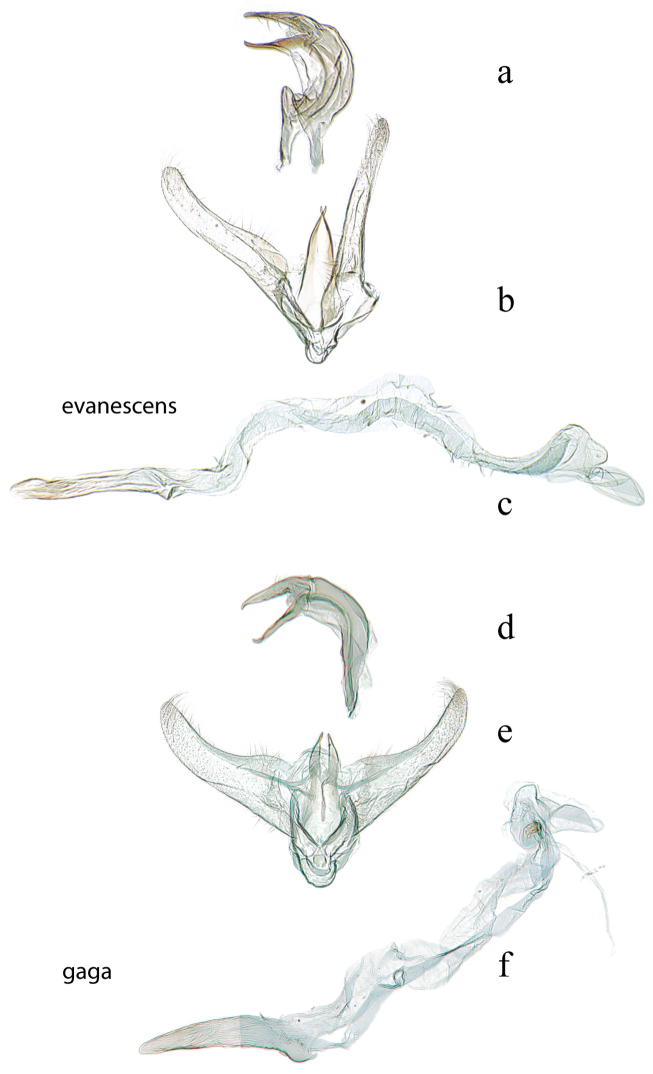
Male genitalia: *Diatraea evanescens*, USNM #114625, Conroe, Texas, USA **a** lateral view uncus, gnathos, tegumen **b** ventral view vinculum, juxta, valvae **c** phallus; *Diatraea gaga*, USNM #114613, El Sombrero, Guarico, Venezuela & USNM #114615, Corazal, Canal Zone, Panama **d** lateral view uncus, gnathos, tegumen **e** ventral view vinculum, juxta, valvae **f** phallus.

**Figure 4.**
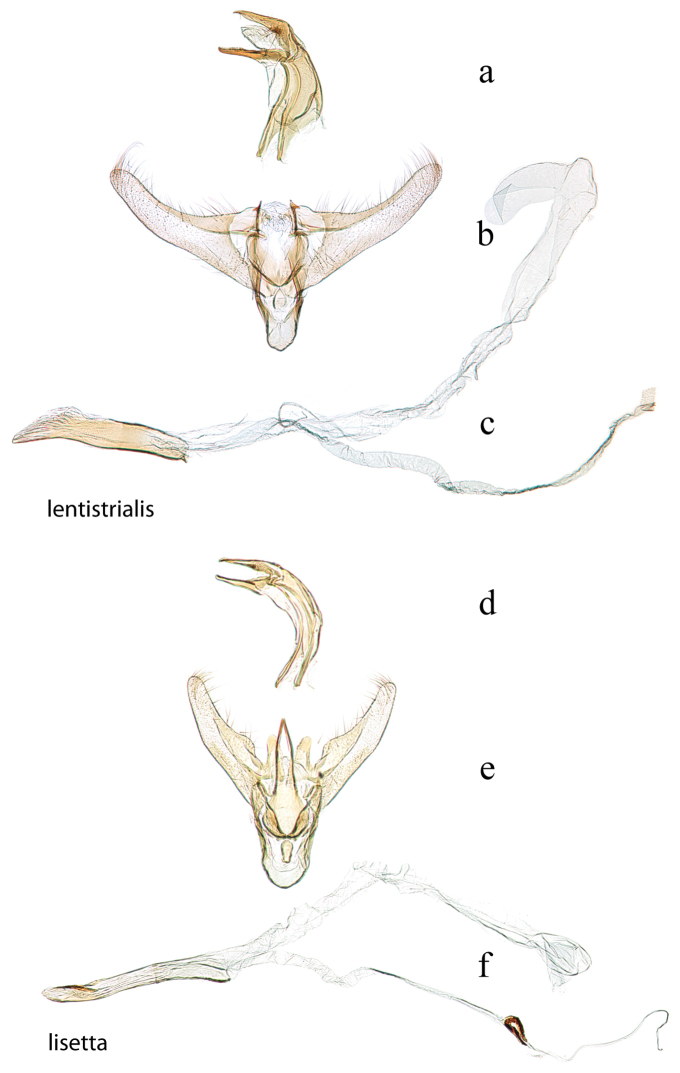
Male genitalia: *Diatraea lentistrialis*, USNM #114616, 30 km. E. of S. Felipe, Yaracuy, Venezuela **a** lateral view uncus, gnathos, tegumen **b** ventral view vinculum, juxta, valvae **c** phallus; *Diatraea lisetta*, holotype, USNM #114618, Dade City, Florida, USA **d** lateral view uncus, gnathos, tegumen **e** ventral view vinculum, juxta, valvae **f** phallus.

**Figure 5.**
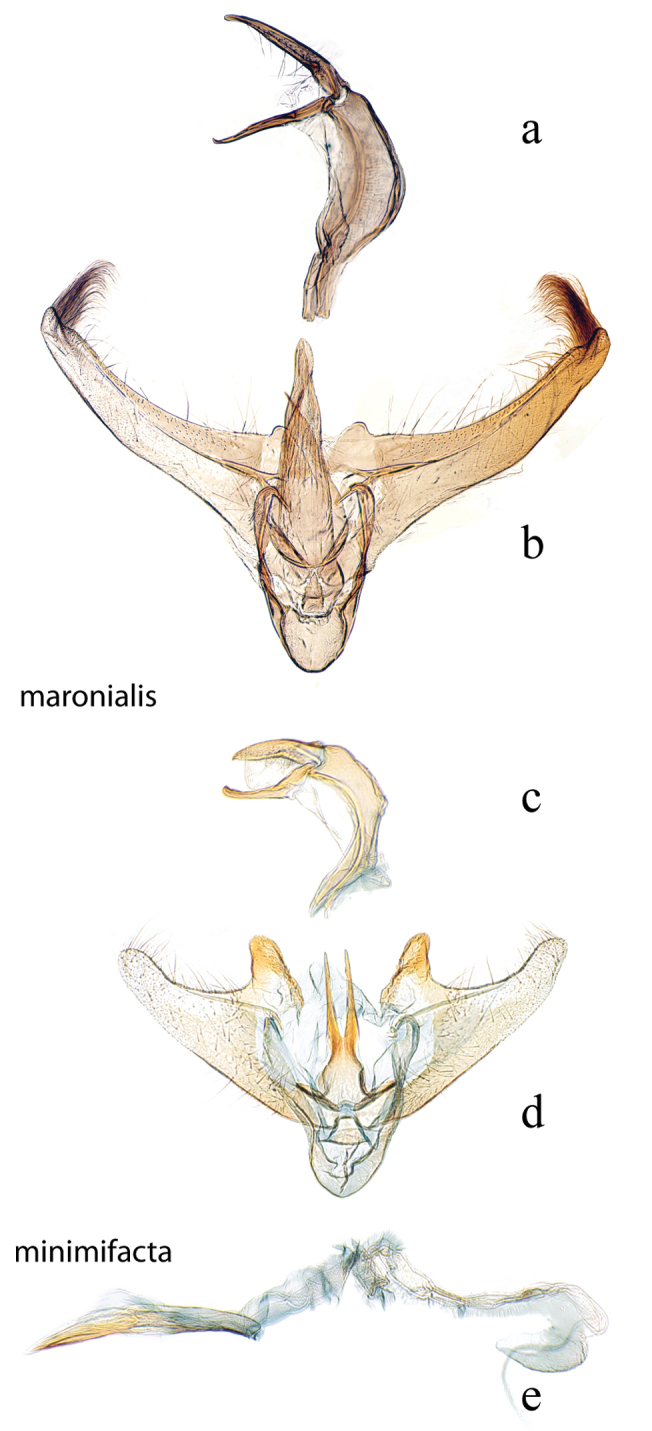
Male genitalia: *Diatraea maronialis*, USNM #97391, St. Jean du Maroni, French Guiana **a** lateral view uncus, gnathos, tegumen **b** ventral view vinculum, juxta, valvae, phallus (attached); *Diatraea minimifacta*, USNM #114621, Yacambu Nat. Pk., Edo. Lara, Venezuela **c** lateral view uncus, gnathos, tegumen **d** ventral view vinculum, juxta, valvae **e** phallus.

**Figure 6.**
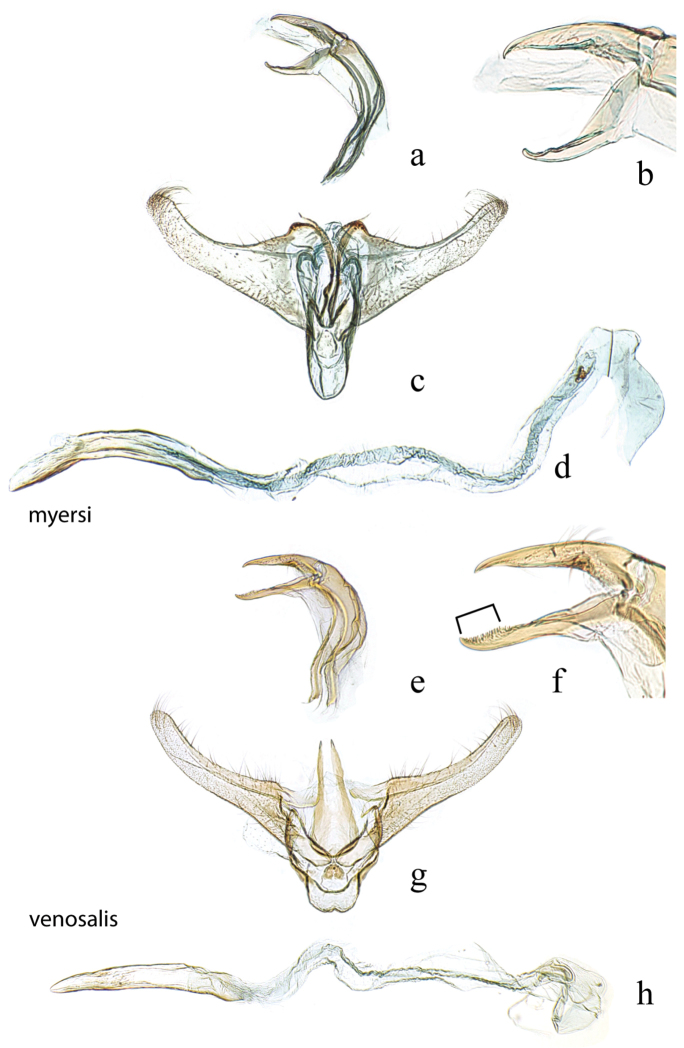
Male genitalia: *Diatraea myersi*, USNM #114622, Rincon National Park, Prov. Guanacaste, Costa Rica **a** lateral view uncus, gnathos, tegumen **b** lateral magnification of gnathos without teeth **c** ventral view vinculum, juxta, valvae **d** phallus; *Diatraea venosalis*, USNM #114623, Bastrop State Park, Bastrop County, Texas, USA **e** lateral view uncus, gnathos, tegumen **f** lateral magnification of gnathos with teeth shown by bracket **g** ventral view vinculum, juxta, valvae **h** phallus.

**Figure 7.**
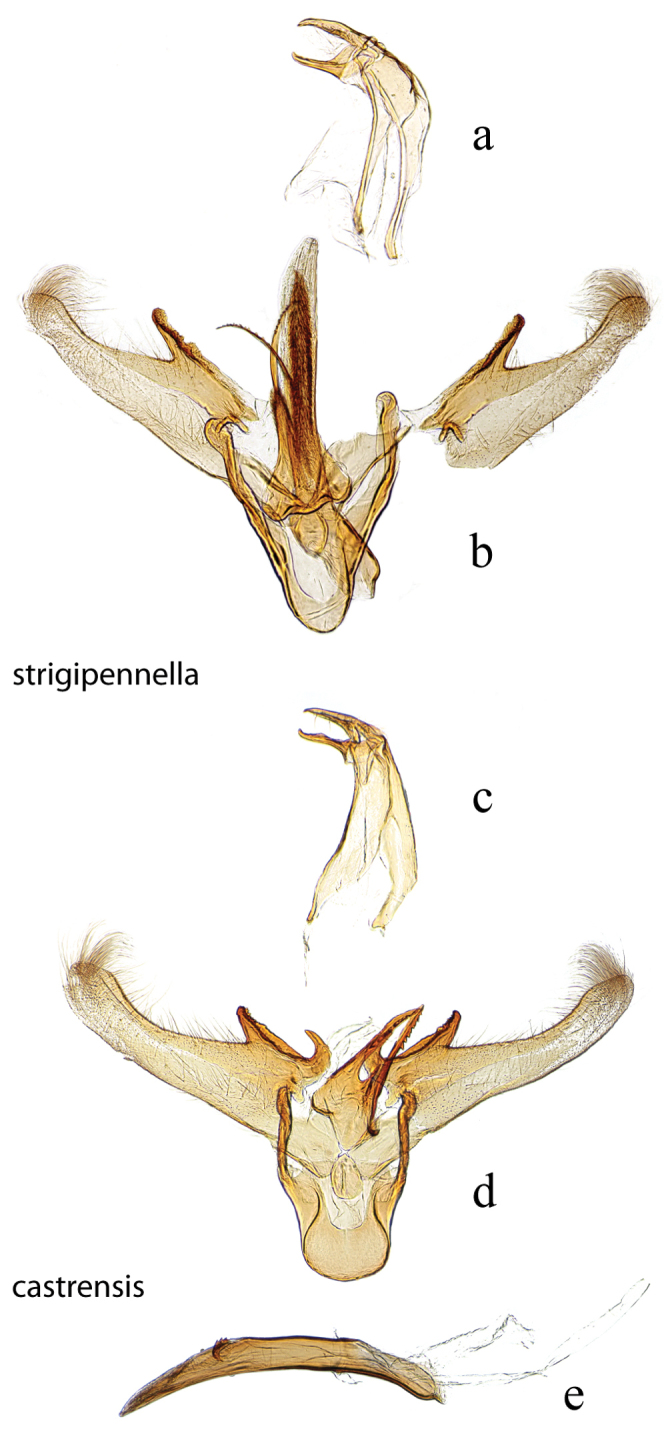
Male genitalia: *Diatraea strigipennella*, USNM #97404, Castro, Parana, Brazil **a** lateral view uncus, gnathos, tegumen **b** ventral view vinculum, juxta, valvae, phallus (attached); *Diatraea castrensis*, USNM #97490, Castro, Parana, Brazil **c** lateral view uncus, gnathos, tegumen **d** ventral view vinculum, juxta, valvae **e** phallus.

**Figure 8.**
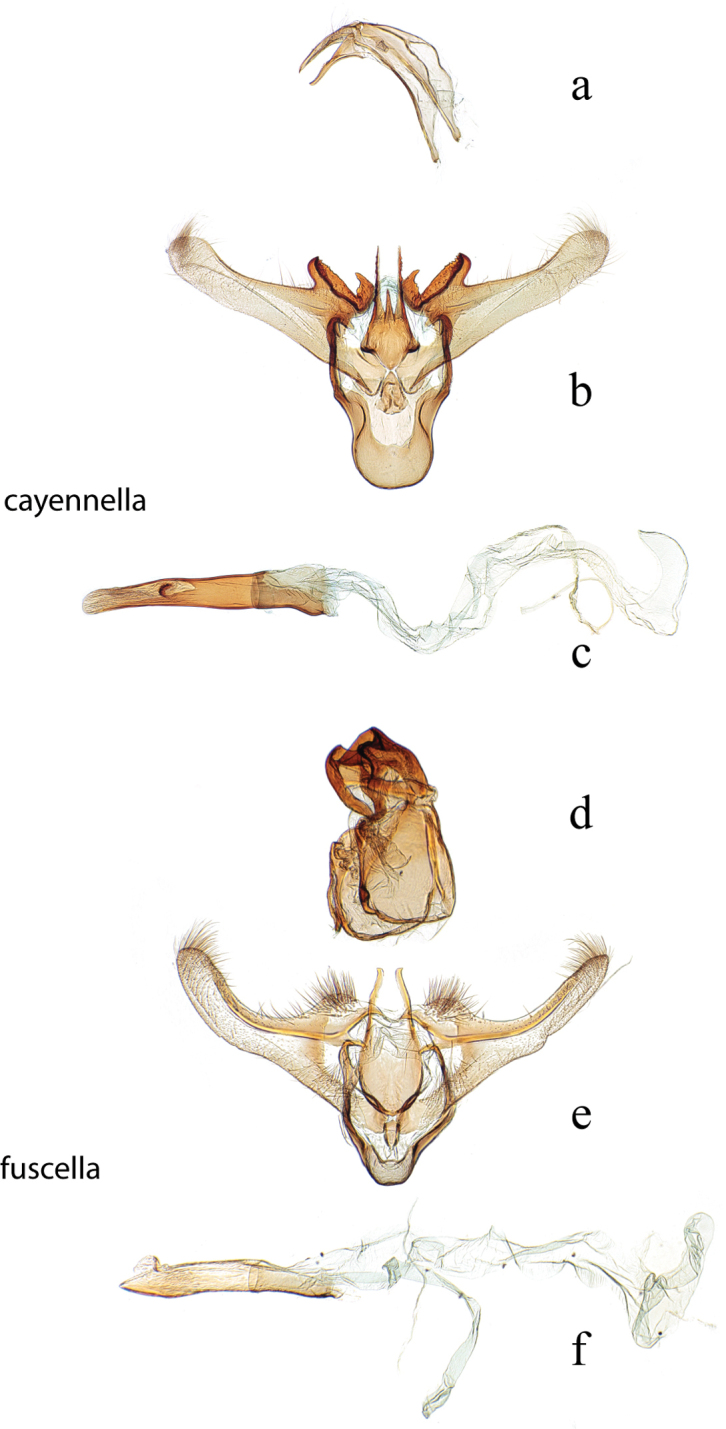
Male genitalia: *Diatraea cayennella*, USNM #114627, Pilcopata, Cuzco, Peru **a** lateral view uncus, gnathos, tegumen **b** ventral view vinculum, juxta, valvae **c** phallus; *Diatraea fuscella*, USNM #114629, Upata, Estacion San Gerardo, Alajuela, Costa Rica **d** lateral view uncus, gnathos, tegumen **e** ventral view vinculum, juxta, valvae **f** phallus.

**Figure 9.**
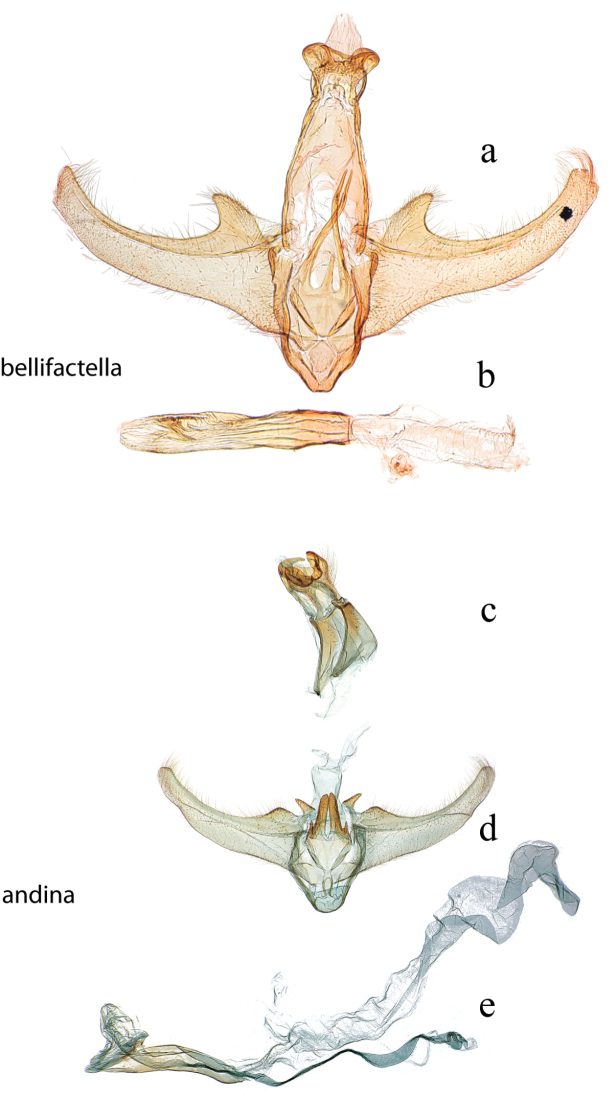
Male genitalia: *Diatraea bellifactella*, USNM #97342, Trinidad, B.W.I. **a** ventral view uncus, gnathos, tegumen, vinculum, juxta, valvae **b** phallus; *Diatraea andina*, USNM #114632, Yacambu Nat. Pk, Edo. Lara, Venezuela **c** lateral view uncus, gnathos, tegumen **d** ventral view vinculum, juxta, valvae **e** phallus.

**Figure 10.**
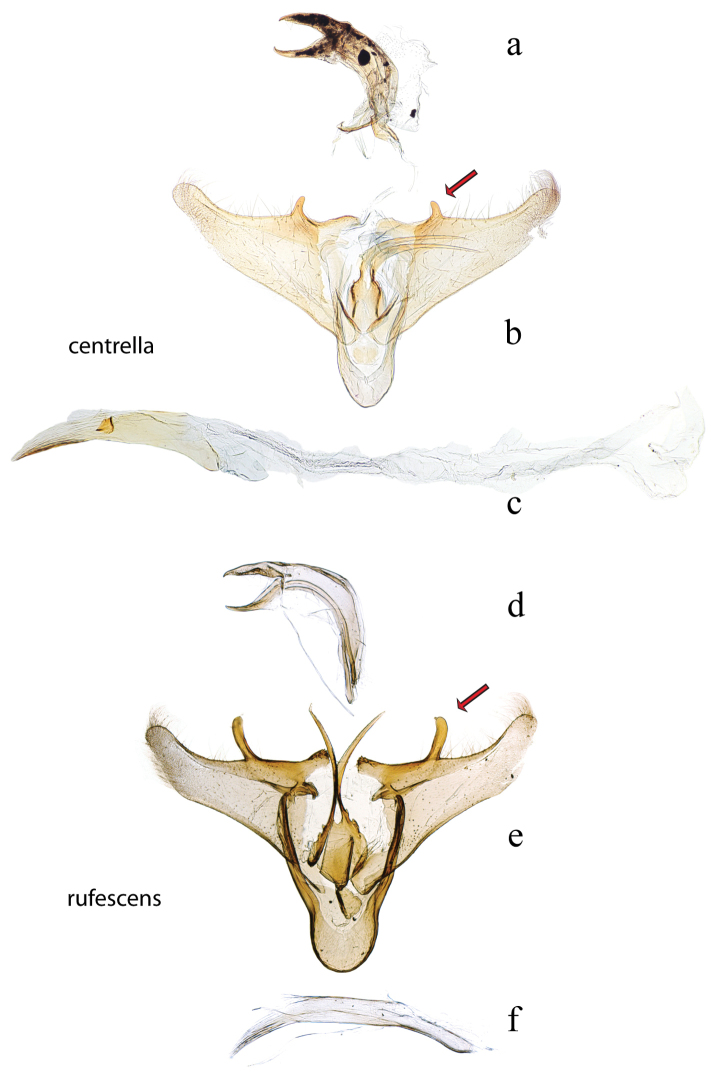
Male genitalia: *Diatraea centrella*, USNM #97425, El Tocuyo, Venezuela **a** lateral view uncus, gnathos, tegumen **b** ventral view vinculum, juxta, valvae, red arrow indicates accessory process **c** phallus; *Diatraea rufescens*, holotype, BMNH #5409, Buenavista, Bolivia **d** lateral view uncus, gnathos, tegumen **e** ventral view vinculum, juxta, valvae, red arrow indicates accessory process **f** phallus.

**Figure 11.**
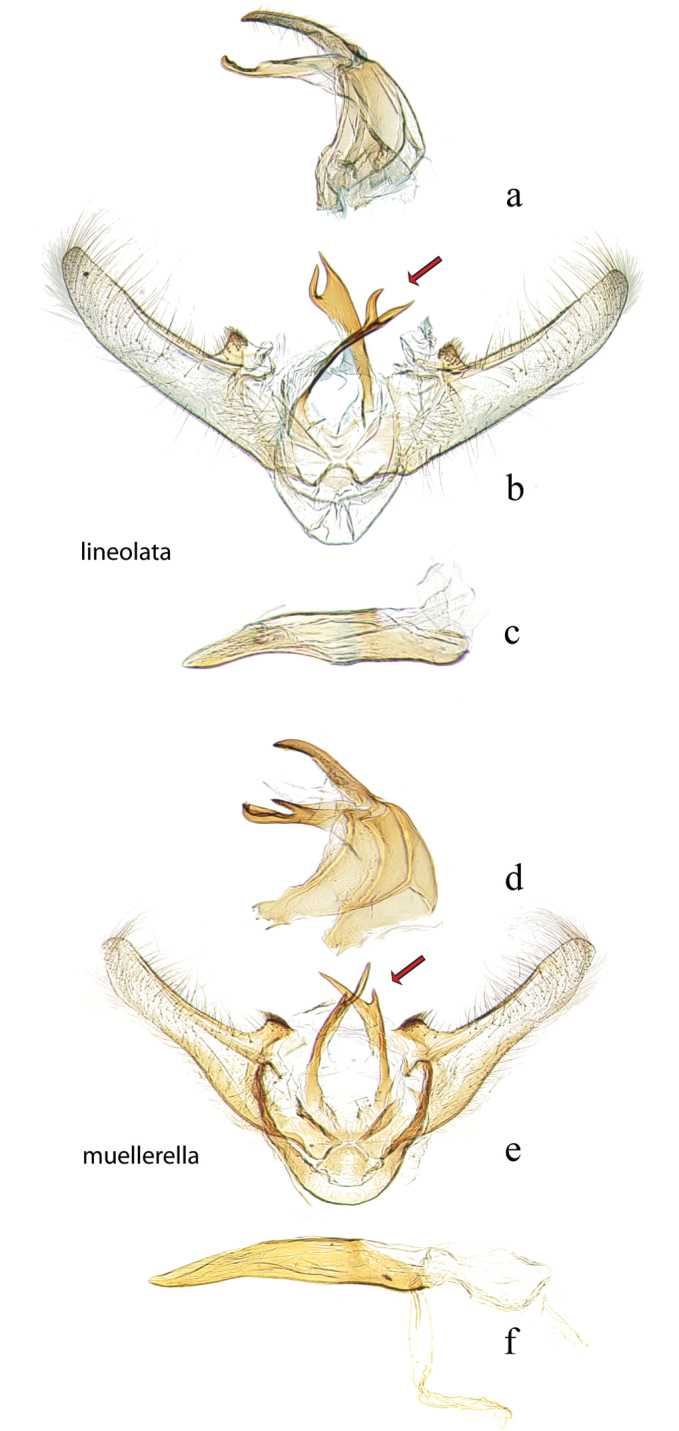
Male genitalia: *Diatraea lineolata*, USNM #113649, Amubri, A.C. Amistad, Prov. Limon, Costa Rica **a** lateral view uncus, gnathos, tegumen **b** ventral view vinculum, juxta, valvae, red arrow indicates juxta **c** phallus; *Diatraea muellerella*, USNM #97436, Iguala, Guerrero, Mexico **d** lateral view uncus, gnathos, tegumen **e** ventral view vinculum, juxta, valvae, red arrow indicates juxta **f** phallus.

**Figure 12.**
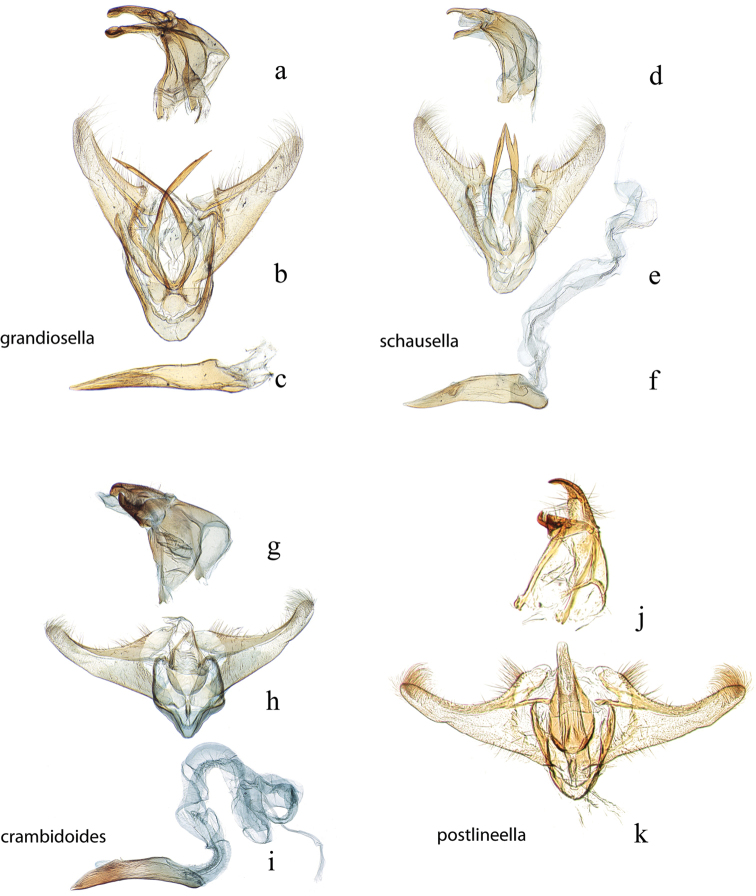
Male genitalia: *Diatraea grandiosella*, USNM #114633, CIMMVT, Mexico **a** lateral view uncus, gnathos, tegumen **b** ventral view vinculum, juxta, valvae **c** phallus; *Diatraea schausella*, USNM #114634, Lancetilia, Honduras **d** lateral view uncus, gnathos, tegumen **e** ventral view vinculum, juxta, valvae **f** phallus.; *Diatraea crambidoides*, USNM #114636, Wedge Plantation, McClellanville, South Carolina, USA **g** lateral view uncus, gnathos, tegumen **h** ventral view vinculum, juxta, valvae **i** phallus; *Diatraea postlineella*, holotype, USNM #97493, Quirigua, Guatemala **j** lateral view uncus, gnathos, tegumen, ventral view vinculum, juxta, valvae **k** phallus.

**Figure 13.**
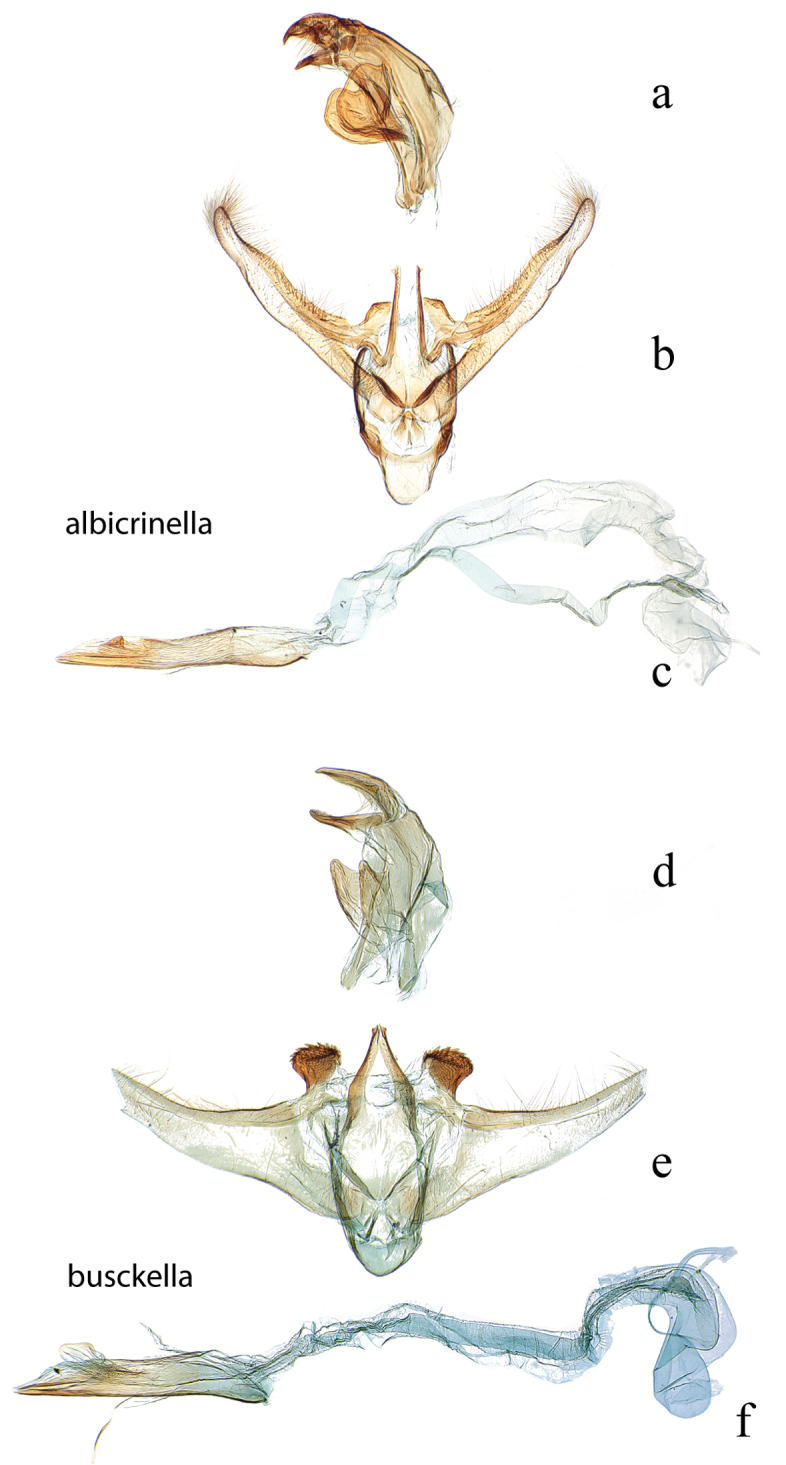
Male genitalia: *Diatraea albicrinella*, USNM #114637, Capitão Poço, PA, Brazil **a** lateral view uncus, gnathos, tegumen **b** ventral view vinculum, juxta, valvae **c** phallus; *Diatraea busckella*, USNM #114640, Baranquilla, Brazil **d** lateral view uncus, gnathos, tegumen **e** ventral view vinculum, juxta, valvae **f** phallus.

**Figure 14.**
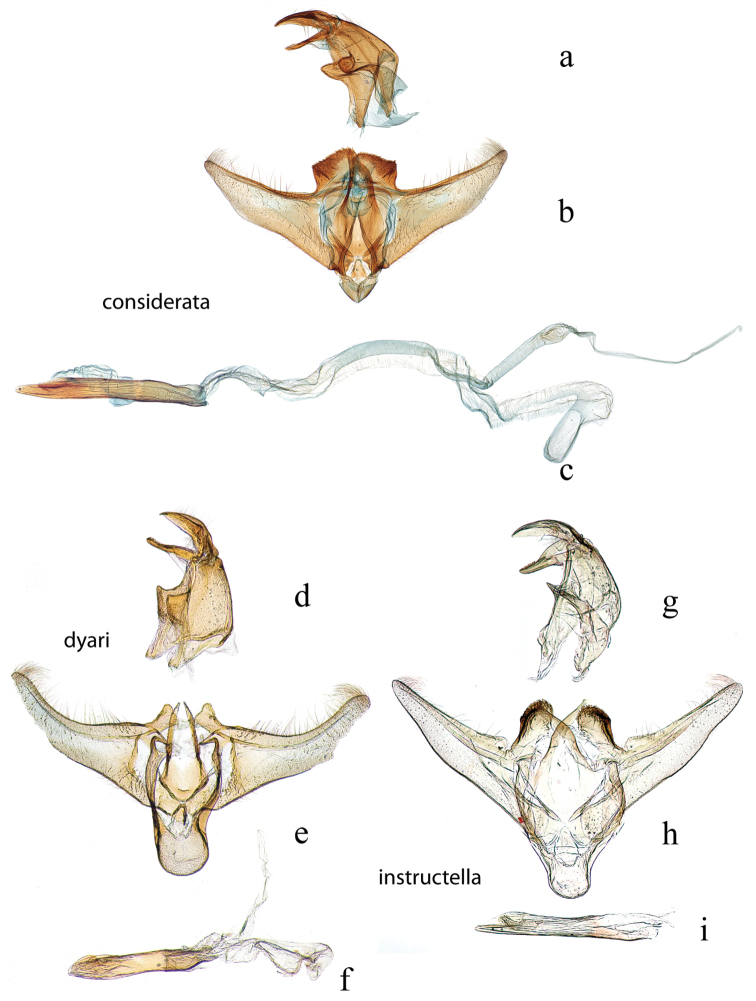
Male genitalia: *Diatraea considerata*, USNM #114642, Villa Union, Sinaloa, Mexico **a** lateral view uncus, gnathos, tegumen **b** ventral view vinculum, juxta, valvae **c** phallus; *Diatraea dyari*, holotype, USNM #22946 **d** lateral view uncus, gnathos, tegumen **e** ventral view vinculum, juxta, valvae **f** phallus; *Diatraea instructella*, USNM #97499, San Jacinto, DF, Mexico **g** lateral view uncus, gnathos, tegumen **h** ventral view vinculum, juxta, valvae **i** phallus.

**Figure 15.**
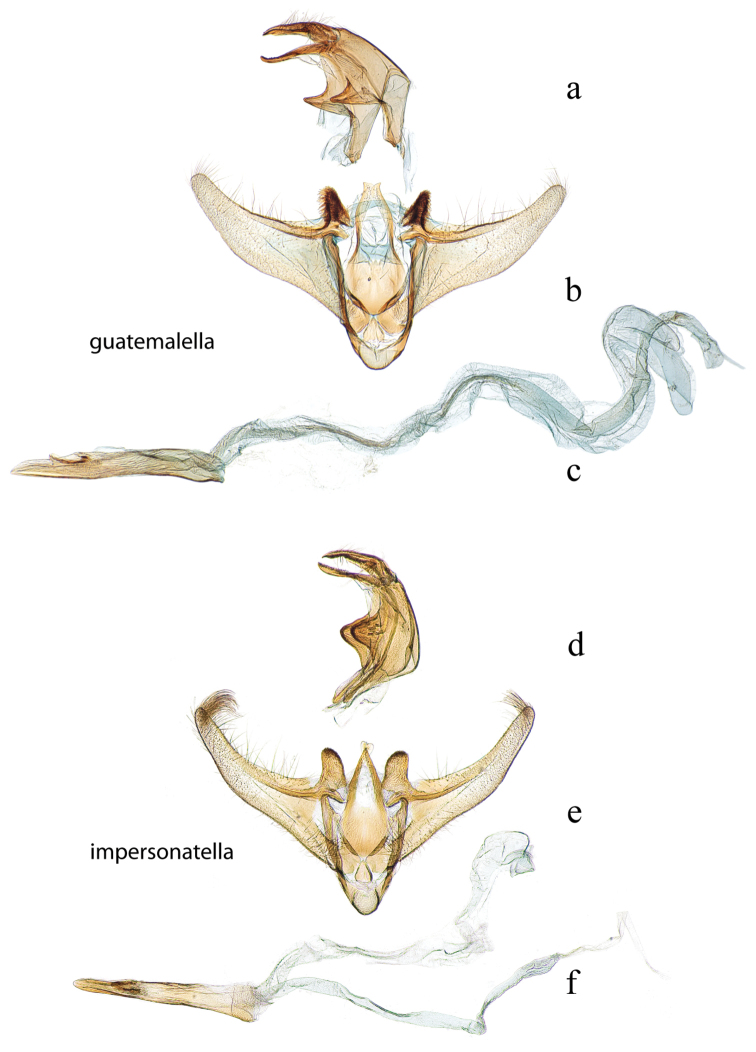
Male genitalia: *Diatraea guatemalella*, USNM #114643, Cayuga, Guatemala **a** lateral view uncus, gnathos, tegumen **b** ventral view vinculum, juxta, valvae **c** phallus; *Diatraea impersonatella*, BMNH #22950, locality unknown, prob. Venezuela (det. Box) **d** lateral view uncus, gnathos, tegumen **e** ventral view vinculum, juxta, valvae **f** phallus.

**Figure 16.**
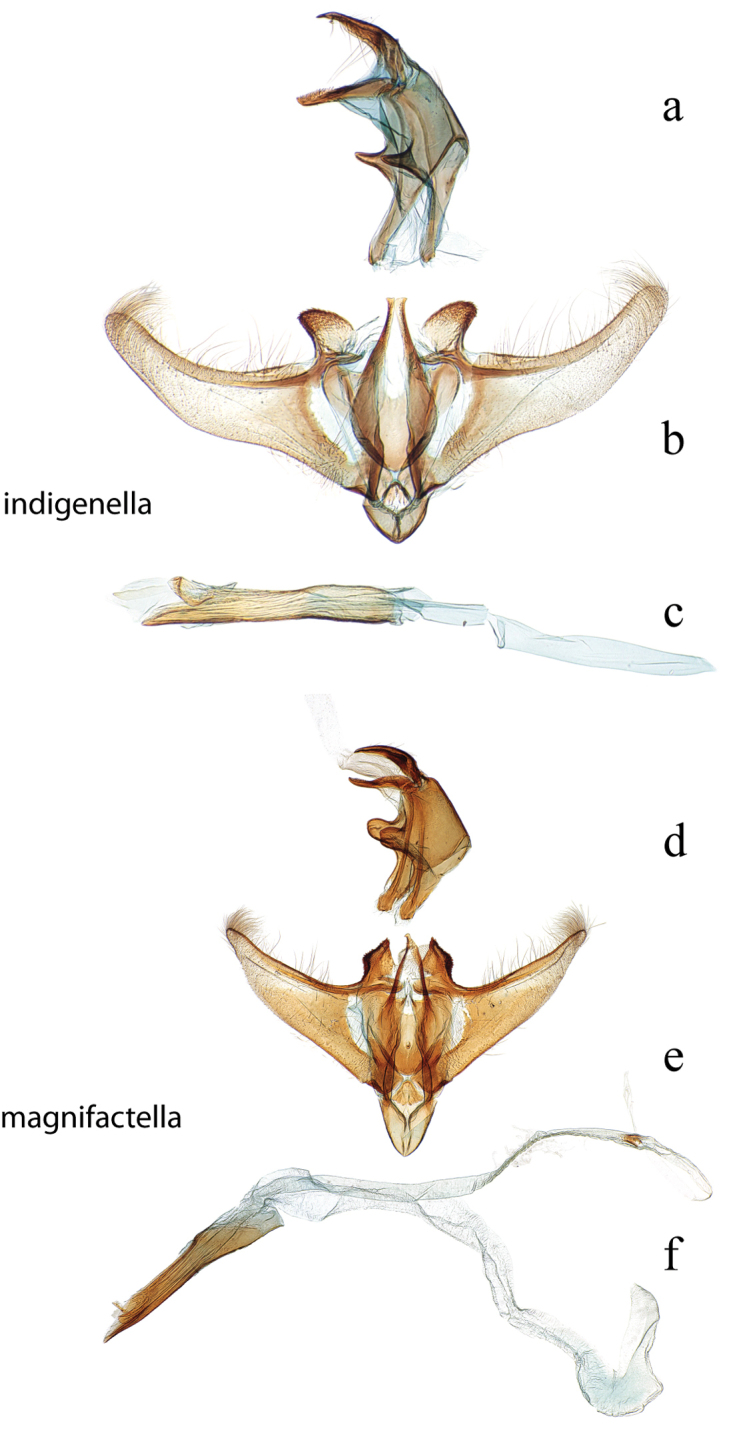
Male genitalia: *Diatraea indiginella*, USNM #114645, Riopaila, Colombia **a** lateral view uncus, gnathos, tegumen **b** ventral view vinculum, juxta, valvae **c** phallus; *Diatraea magnifactella*, USNM #114646, near Jalapa, Veracruz, Mexico **d** lateral view uncus, gnathos, tegumen **e** ventral view vinculum, juxta, valvae **f** phallus.

**Figure 17.**
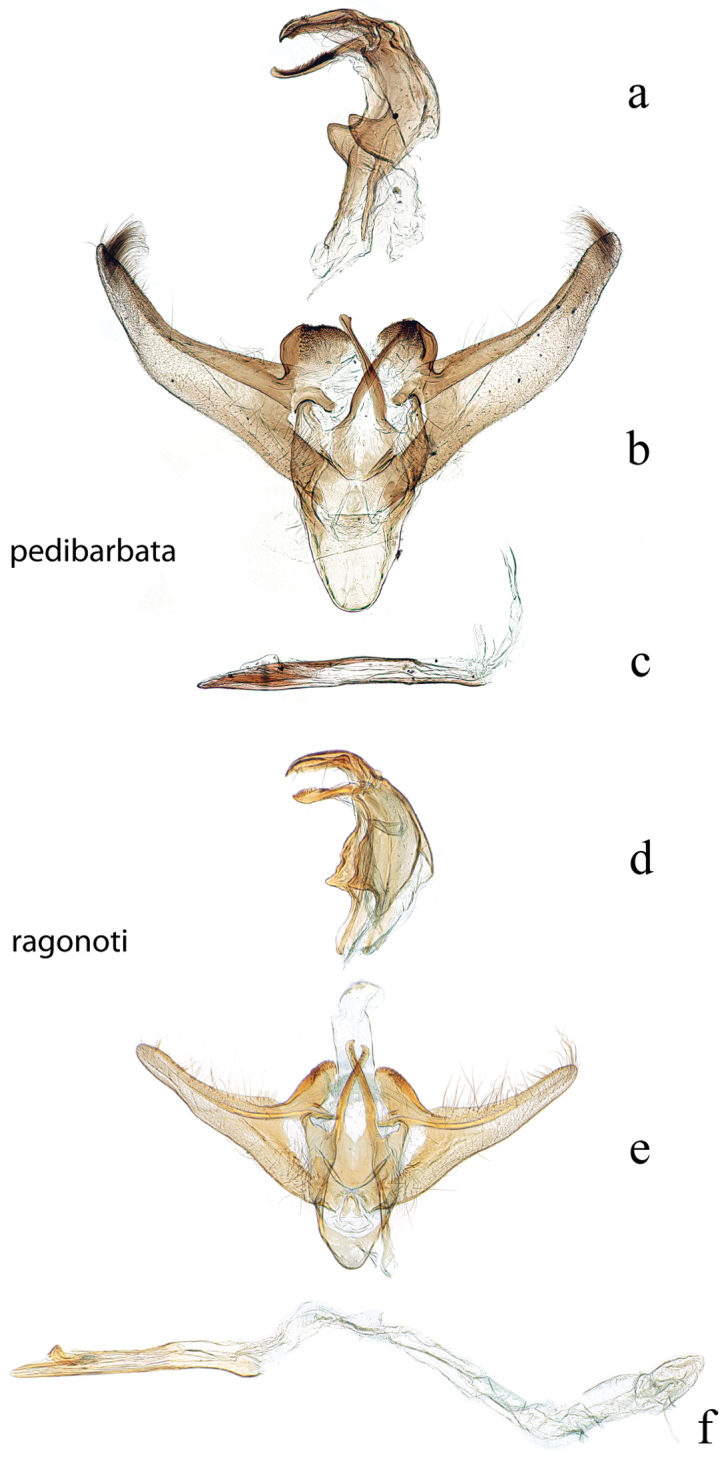
Male genitalia: *Diatraea pedibarbata*, USNM #97322, Venezuela **a** lateral view uncus, gnathos, tegumen **b** ventral view vinculum, juxta, valvae **c** phallus; *Diatraea ragonoti*, USNM #114648, Petropolis, Brazil **d** lateral view uncus, gnathos, tegumen **e** ventral view vinculum, juxta, valvae **f** phallus.

**Figure 18.**
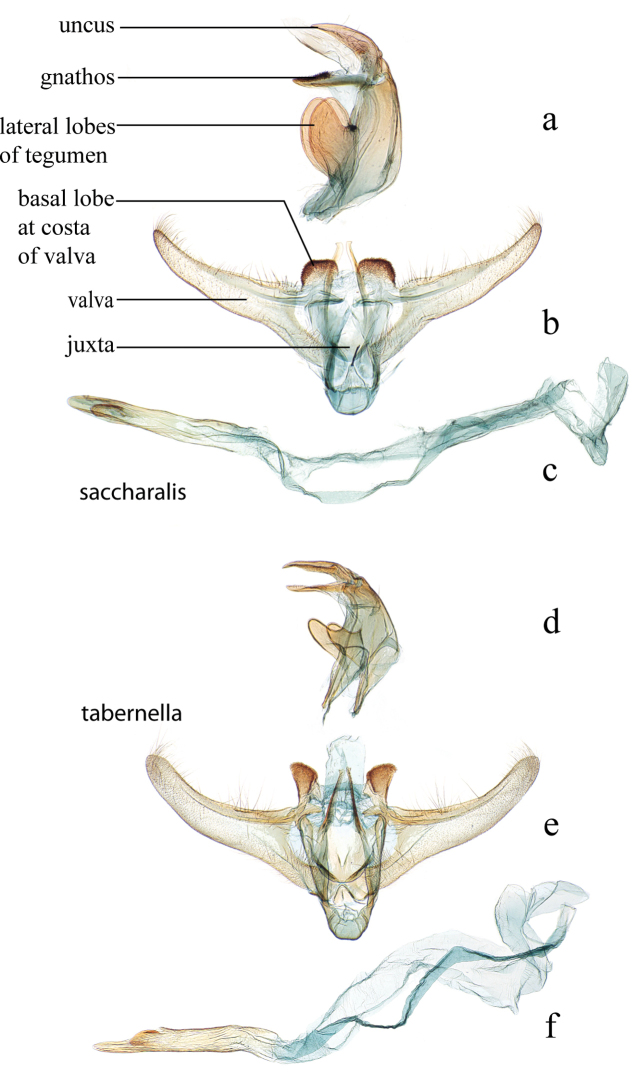
Male genitalia: *Diatraea saccharalis*, USNM #114649, Deutschburg, Jackson Co., Texas, USA **a** lateral view uncus, gnathos, tegumen **b** ventral view vinculum, juxta, valvae **c** phallus; *Diatraea tabernella*, USNM #114654, Rio Trinidad, Panama, #114656, Rio Trinidad, Panama **d** lateral view uncus, gnathos, tegumen **e** ventral view vinculum, juxta, valvae **f** phallus.

**Figure 19.**
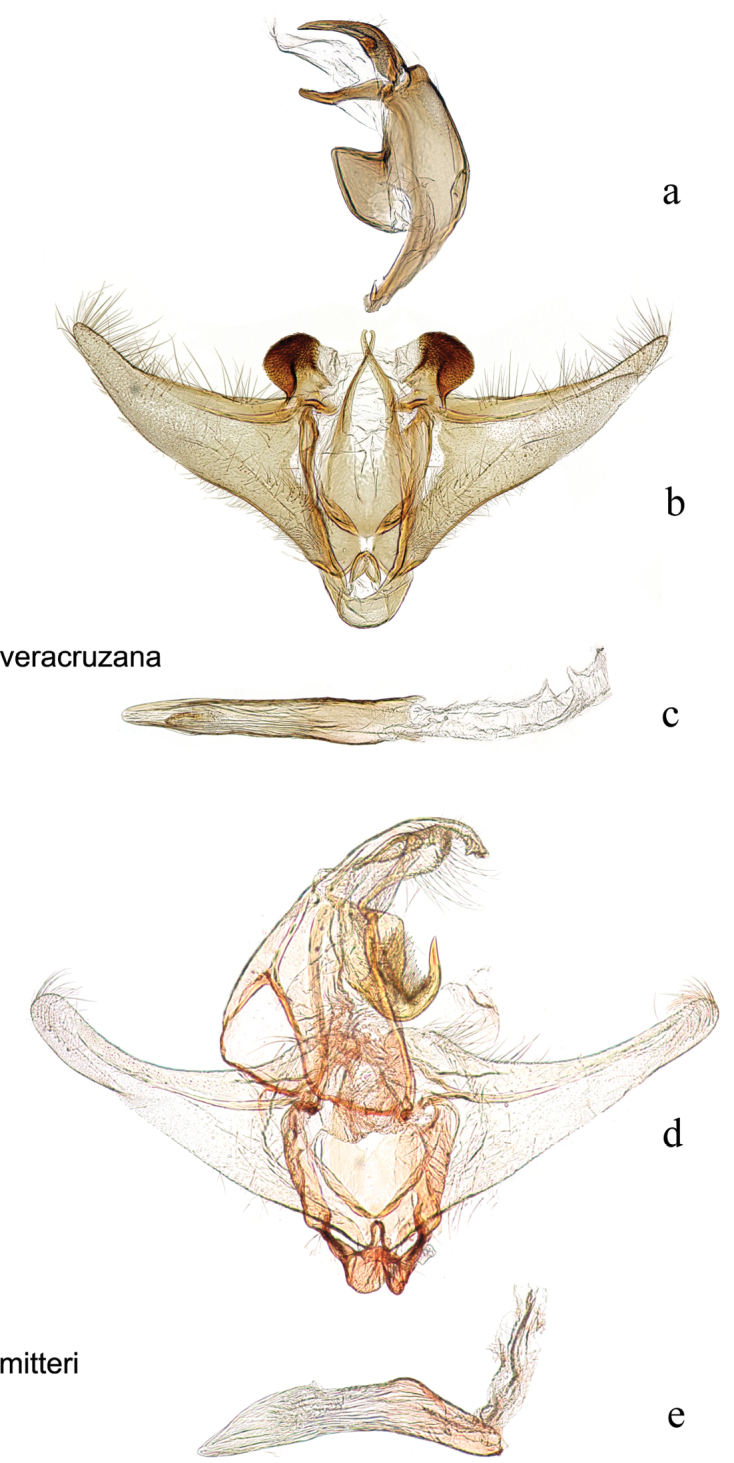
Male genitalia: *Diatraea veracruzana*, USNM #97476, nr. San Cristobal, Veracruz, Mexico **a** lateral view uncus, gnathos, tegumen **b** ventral view vinculum, juxta, valvae **c** phallus; *Diatraea mitteri*, USNM #97234, Churchill, Texas, USA **d** ventral view uncus, gnathos, tegumen, vinculum, juxta, valvae **e** phallus.

**Figure 20.**
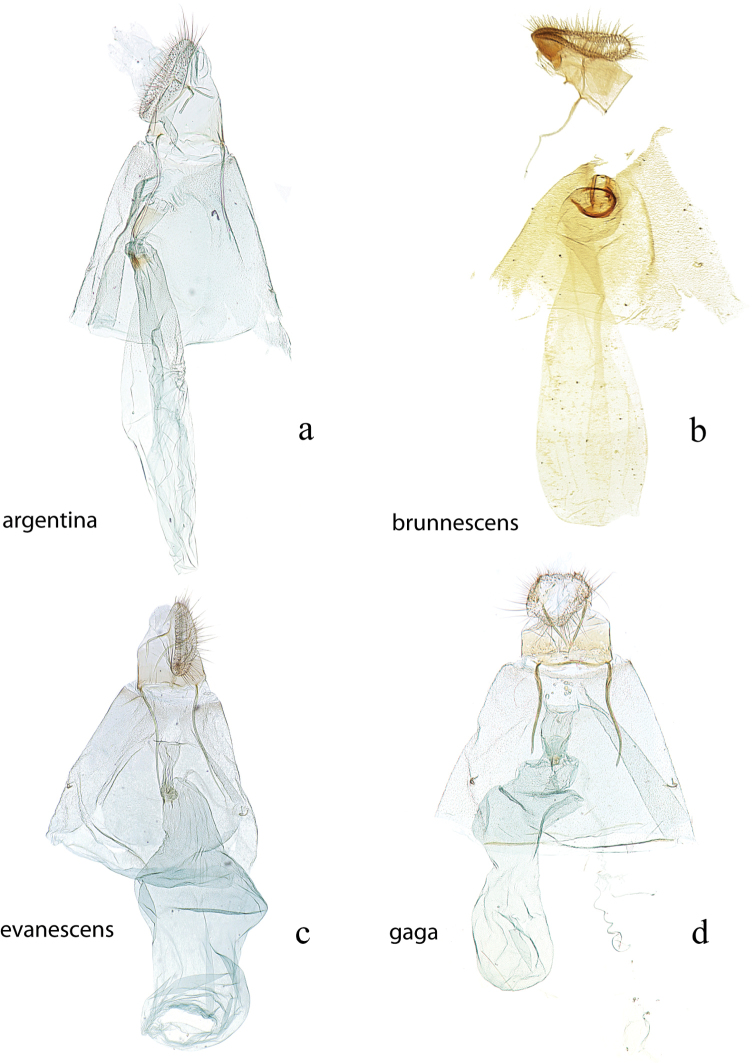
Female genitalia, ventral view: **a** *Diatraea argentina*, CMNH #004, Santa Cruz, Provincia del Sara, Bolivia **b** *Diatraea brunnescens*, paratype, BMNH #120, Ciudad Bolivar, Venezuela **c** *Diatraea evanescens*, USNM #114624, Camp Strake, Montgomery Co, Texas, USA **d** *Diatraea gaga*, USNM #114614, El Sombrero, Guarico, Venezuela.

**Figure 21.**
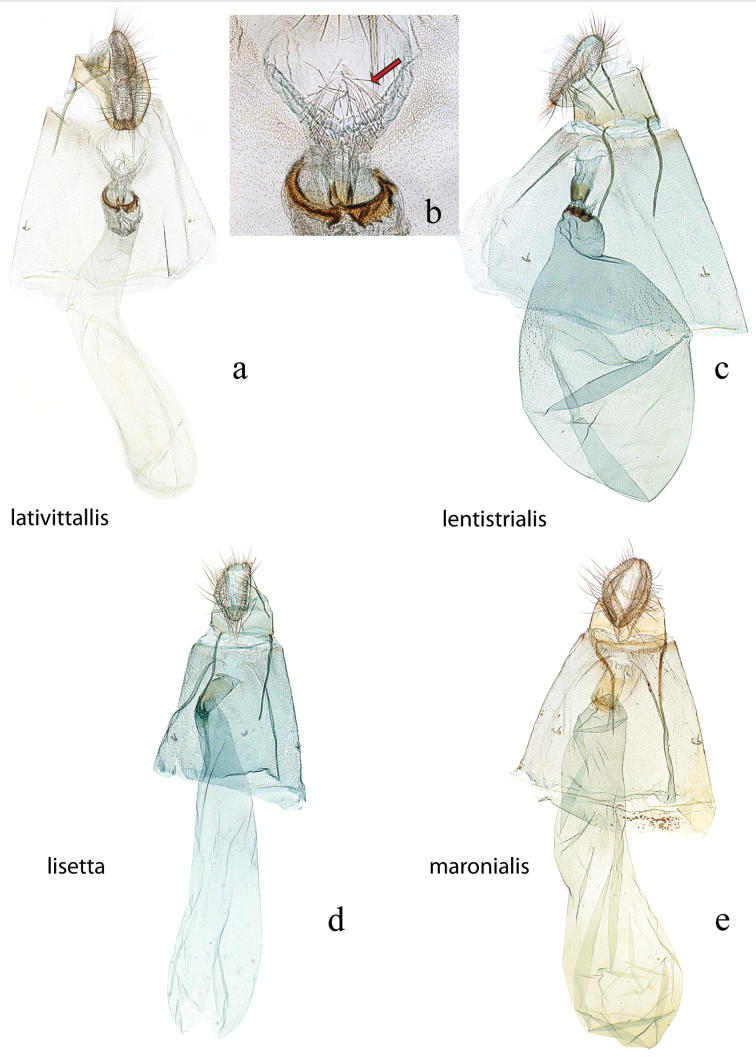
Female genitalia, ventral view: **a** *Diatraea lativittalis*, CMNH #024, Puerto Suarez, Bolivia **b** magnification of setae near ostium bursae shown by red arrow **c** *Diatraea lentistrialis*, USNM #114617, Santa Rosa National Park, Prov. Guanacaste, Costa Rica **d** *Diatraea lisetta*, USNM #114619, Oneco, Manatee Co., Florida, USA **e** *Diatraea marionalis*, USNM #114620, St. Jean du Maroni, French Guiana.

**Figure 22.**
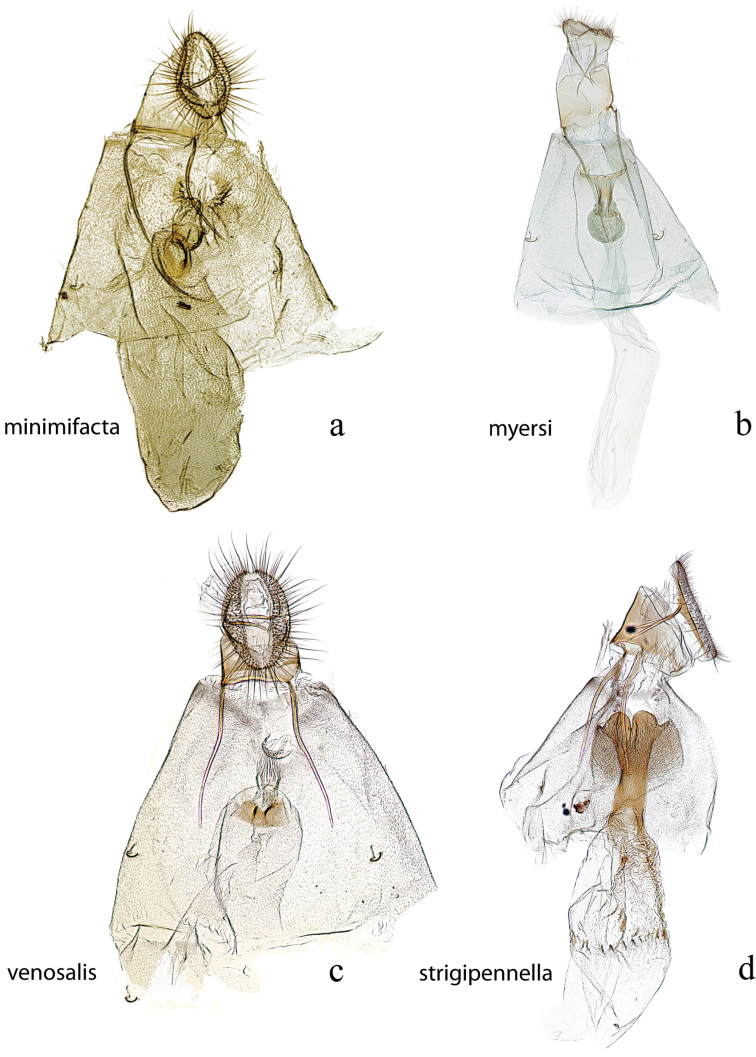
Female genitalia, ventral view: **a** *Diatraea minimifacta*, syntype, USNM #99604, Trinidad, British West Indies **b** *Diatraea myersi*, CMNH #003, Obidos, Brazil **c** *Diatraea venosalis*, USNM #111879, Audubon Park, Louisiana, USA **d** *Diatraea strigipennella*, USNM #97403, Baboquivari Mts., Arizona, USA.

**Figure 23.**
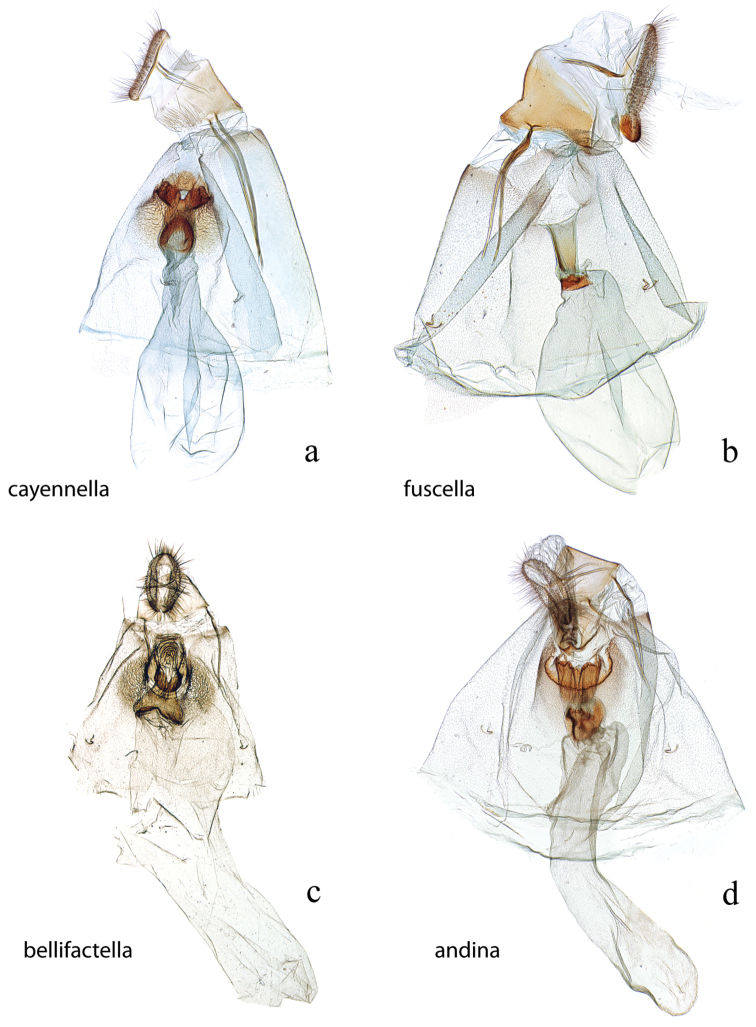
Female genitalia, ventral view: **a** *Diatraea cayennella*, USNM #114628, Pilcopata, Cuzco, Peru **b** *Diatraea fuscella*, USNM #114630, Estacion San Gerardo, Alajuela, Costa Rica **c** *Diatraea bellifactella*, syntype, USNM #97339, Castro, Parana, Brazil **d** *Diatraea andina*, USNM #114631, Portuguesa Alto, Venezuela.

**Figure 24.**
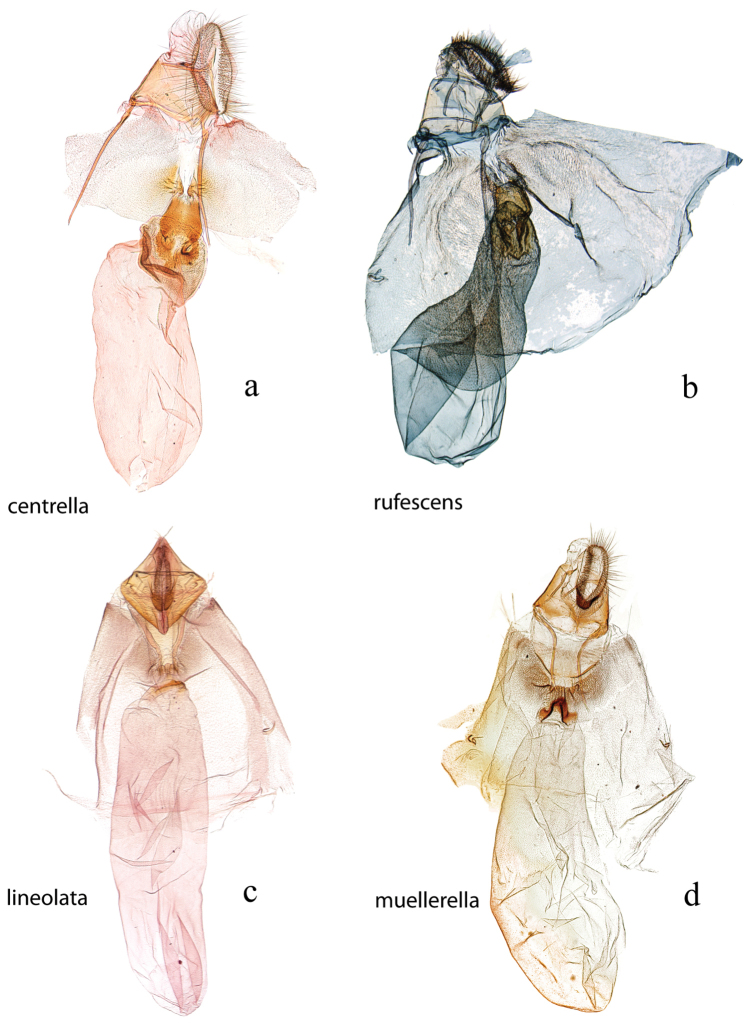
Female genitalia, ventral view: **a** *Diatraea centrella*, USNM #97415, Itacoatiara, Amazon **b** *Diatraea rufescens*, BMNH #17084, Santa Cruz, Bolivia **c** *Diatraea lineolata*, BMNH #10724, Venezuela **d** *Diatraea muellerella*, USNM #97435, Iguala, Mexico.

**Figure 25.**
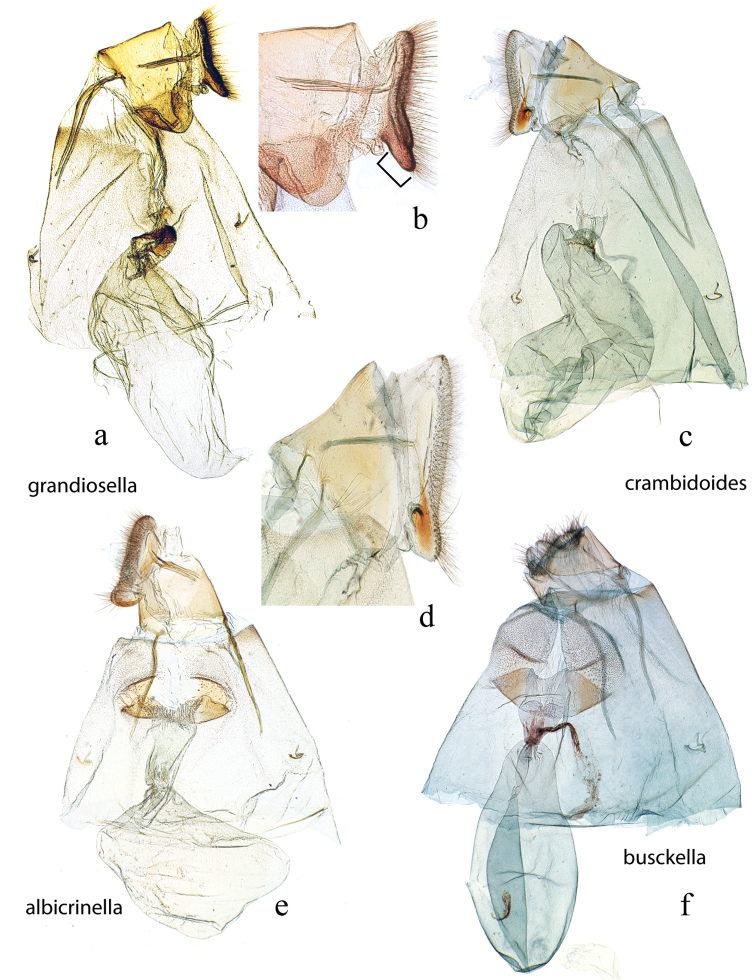
Female genitalia, ventral view: **a** *Diatraea grandiosella*, holotype, USNM #97390, Guadalajara, Mexico **b** magnification of papillae analis with lobe-like ventral extension shown by bracket **c** *Diatraea crambidoides*, USNM #114635, Wedge Plantation, McClellanville, South Carolina, USA **d** magnification of papillae analis without lobe-like ventral extension **e** *Diatraea albicrinella*, USNM #114638, Capitão Poço, PA, Brazil **f** *Diatraea busckella*, USNM #114639, Baranquilla, Brazil.

**Figure 26.**
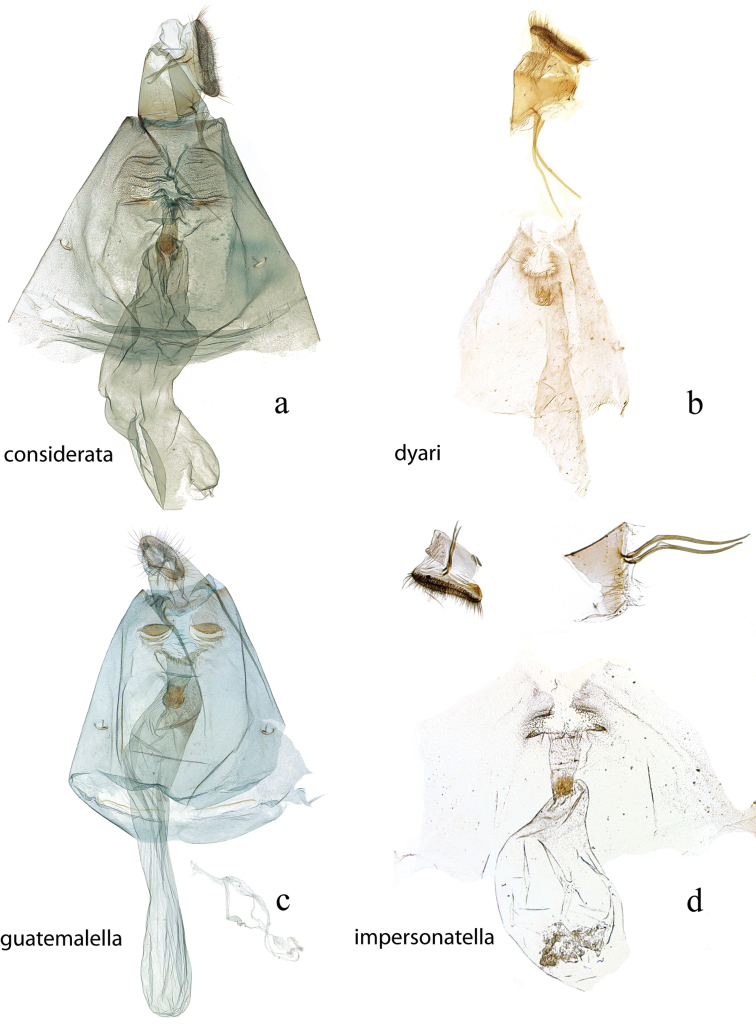
Female genitalia, ventral view: **a** *Diatraea considerata*, USNM #114641, Villa Union, Sinaloa, Mexico **b** *Diatraea dyari*, paratype, BMNH #53, Argentina (disarticulated) **c** *Diatraea guatemalella*, USNM #114644, Cayuga, Guatemala **d** *Diatraea impersonatella* (disarticulated), BMNH #142, Trinidad.

**Figure 27.**
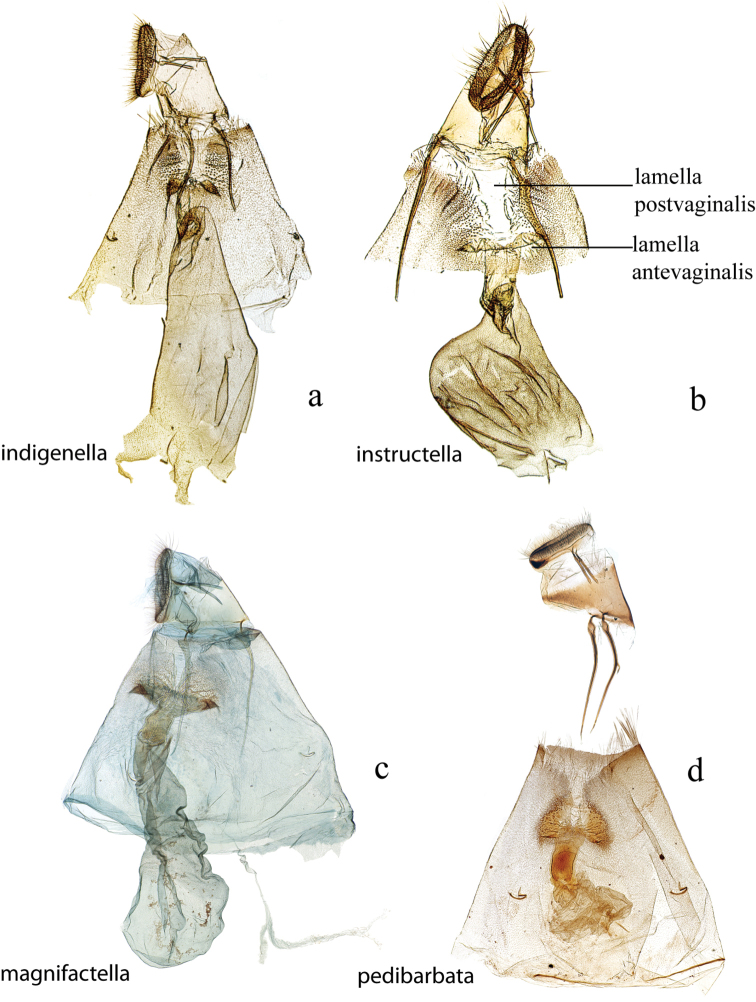
Female genitalia, ventral view: **a** *Diatraea indigenella*, USNM #97389, Papayan, Colombia **b** *Diatraea instructella*, holotype, USNM #97498, Popocatepetl, Mexico **c** *Diatraea magnifactella*, USNM #114647, near Jalapa, Veracruz, Mexico **d** *Diatraea pedibarbata*, USNM #97323, Venezuela.

**Figure 28.**
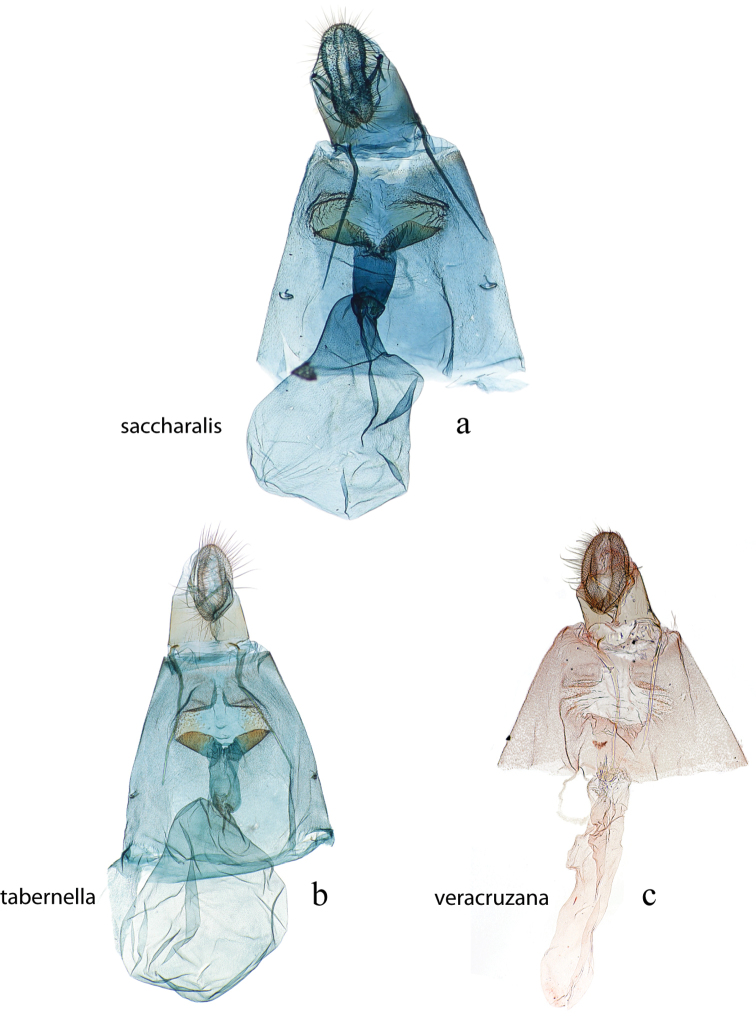
Female genitalia, ventral view: **a** *Diatraea saccharalis*, USNM #115309, Unaí, MG, Brazil **b** *Diatraea tabernella*, USNM #114655, Rio Trinidad, Panama **c** *Diatraea veracruzana*, USNM #97477, nr. San Cristobal, Veracruz, Mexico.

**Figure 29.**
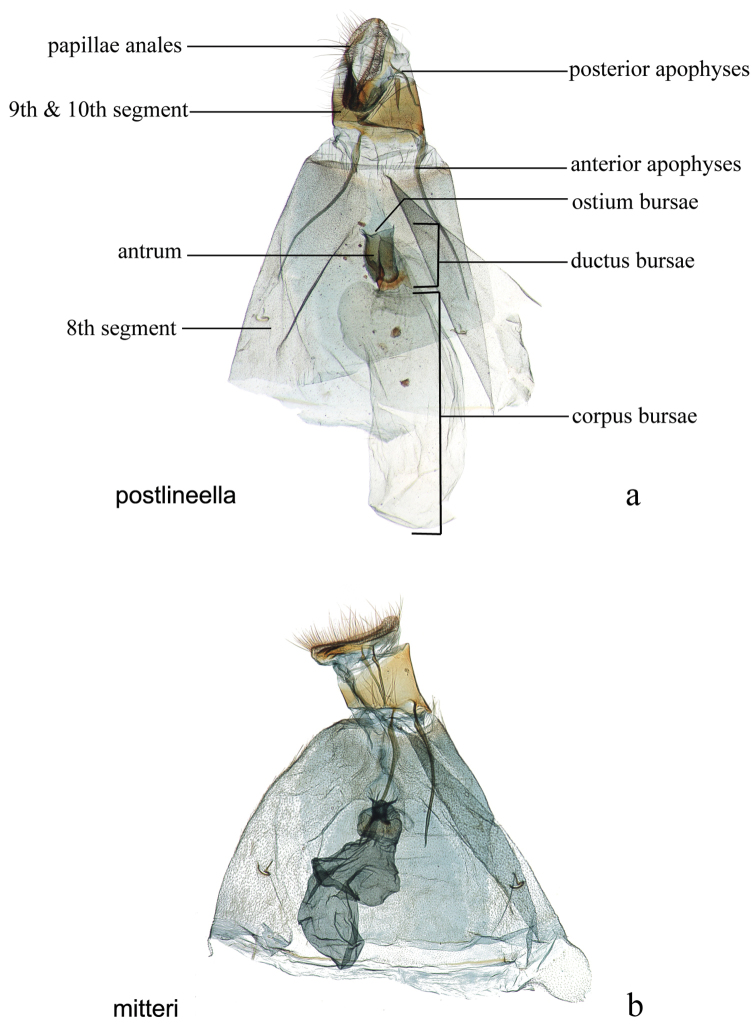
Female genitalia, ventral view: **a** *Diatraea postlineella*, USNM #115510, Escuintla, Km. 64.5 Ca St. Lucia Cotz, Guatemala **b** *Diatraea mitteri*, USNM #112892, Woodward, Oklahoma, USA.

## Supplementary materials

Supplementary material 1 Distribution of *Diatraea* species by country based on Box (1931) and the USNM collection. Data type: xls file Explanation note: Matrix of species of *Diatraea* and countries by subregion of the Western Hemisphere This dataset is made available under the Open Database License (http://opendatacommons.org/licenses/odbl/1.0/). The Open Database License (ODbL) is a license agreement intended to allow users to freely share, modify, and use this Dataset while maintaining this same freedom for others, provided that the original source and author(s) are credited.M. Alma Solis, Mark A. Metz
